# The WinPro trial: window-of-opportunity study of endocrine therapy with micronised progesterone in ER-positive breast cancer

**DOI:** 10.1038/s41523-026-01008-w

**Published:** 2026-07-21

**Authors:** Lucy Haggstrom, Kate Middleton, Andrew Parker, Davendra Segara, Andrew Ong, Janne Bingham, Emma-Kate Carson, Belinda. E. Kiely, Dhanusha Sabanathan, Andrew Spillane, Kate Saw, Aura Serrano, Geoffrey J. Lindeman, Alexander Swarbrick, Alan Coates, G. Bruce Mann, Wayne Tilley, Elgene Lim

**Affiliations:** 1 St Vincent’s Hospital, Sydney, NSW Australia; 2 St Vincent’s Clinical School, The University of New South Wales, Sydney, NSW Australia; 3 Garvan Institute of Medical Research, Sydney, NSW Australia; 4 Department of Medical Oncology, Campbelltown Hospital, Sydney, NSW Australia; 5 Department of Surgery, Royal Adelaide Hospital, Adelaide, SA Australia; 6 Nepean Cancer Care Centre, Nepean Hospital, Sydney, NSW Australia; 7 The Mater Hospital, Sydney, NSW Australia; 8 The University of Sydney, Sydney, NSW Australia; 9 Royal North Shore Hospital, St Leonards, NSW Australia; 10 Peter MacCallum Cancer Care Centre, Melbourne, VIC Australia; 11 The Walter and Eliza Hall Institute of Medical Research, Melbourne, VIC Australia; 12 The Royal Melbourne Hospital, Parkville, VIC Australia; 13 Dame Roma Mitchell Cancer Research Laboratory, University of Adelaide, Adelaide, SA Australia

**Keywords:** Cancer, Diseases, Drug discovery, Medical research, Oncology

## Abstract

Progesterone can inhibit tumoural proliferation in oestrogen receptor positive (ER+) breast cancer. The WinPro trial (Australian New Zealand Trials Clinical Trials Registry number: ACTRN12618000928213, date of registration 01/06/2018) was a randomised, multi-centre, phase 2, window-of-opportunity trial evaluating micronised progesterone (MP) in post-menopausal women with early-stage, ER+, progesterone receptor (PR) positive, HER2- breast cancer. Patients were randomised to letrozole, letrozole and MP, or tamoxifen and MP for 14 days preoperatively. The primary endpoint was the percent proportional reduction in Ki67 (‘Ki67 suppression’) between the two letrozole groups in the per protocol population. From February 2018 to June 2024, 244 patients were enrolled. 189 patients completed per protocol: letrozole (*n* = 66, 34.9%), letrozole + MP (*n* = 64, 33.9%), and tamoxifen + MP (*n* = 59, 31.2%). There was no difference in Ki67 suppression between letrozole (88.2%) versus letrozole + MP (89.2%) (*p* = 0.39). Ki67 suppression appeared lower with tamoxifen + MP (61.5%). Hot flushes appeared less frequent with letrozole + MP (13.3%) versus letrozole (22.4%) or tamoxifen + MP (20.5%).

## Introduction

Oestrogen receptor positive (ER+) breast cancer accounts for approximately 75% of all breast cancers^1^, and despite significant improvements in treatment over the last several decades, up to 30% of high-risk patients will relapse^2^. Furthermore, endocrine therapy, which is the foundation of treatment of ER+ breast cancer, is poorly tolerated and up to 50% of women cease endocrine therapy before the recommended duration, leading to poorer outcomes^3–6^. There is an urgent need to improve outcomes for patients with ER+ breast cancer by developing novel therapeutic strategies and improving tolerance of and adherence to established therapies. Window of opportunity studies are a clinical trial design that are useful for rapidly evaluating the effect of novel therapeutics, as the change in Ki67 after short-term preoperative endocrine therapy is prognostic and a surrogate for long-term outcomes^7^.

Preclinical evidence has demonstrated that progesterone is a tumour suppressor in oestrogen receptor (ER+) positive breast cancer^8^. The progesterone receptor (PR) interacts with ER to reprogram where it binds to chromatin, leading to upregulation of genes associated with improved prognosis^8^. Synthetic progestogens (i.e. progestins), such as megestrol acetate and medroxyprogesterone acetate, were historically used at high doses to treat advanced breast cancer, with some studies showing that response rates and overall survival were superior to tamoxifen^9,10^. A recent window of opportunity study evaluated the addition of megestrol acetate to letrozole in postmenopausal women with ER+, HER2- breast cancer and demonstrated that the addition of megestrol acetate improved Ki67 suppression compared to letrozole alone^11^. We aimed to determine whether a pure PR agonist could be effective in combination with endocrine therapy in ER+ breast cancer. Micronised progesterone (MP, Prometrium) is chemically identical to progesterone of human ovarian origin, a pure PR ligand, and is currently licensed for use as menopausal hormone therapy^12,13^. We conducted the WinPro trial (ACTRN12618000928213), a phase 2 randomised window of opportunity study investigating the addition of MP to endocrine therapy in postmenopausal patients with ER+, PR+, HER2- early stage breast cancer.

## Results

### Enrolment

**Fig. 1:**
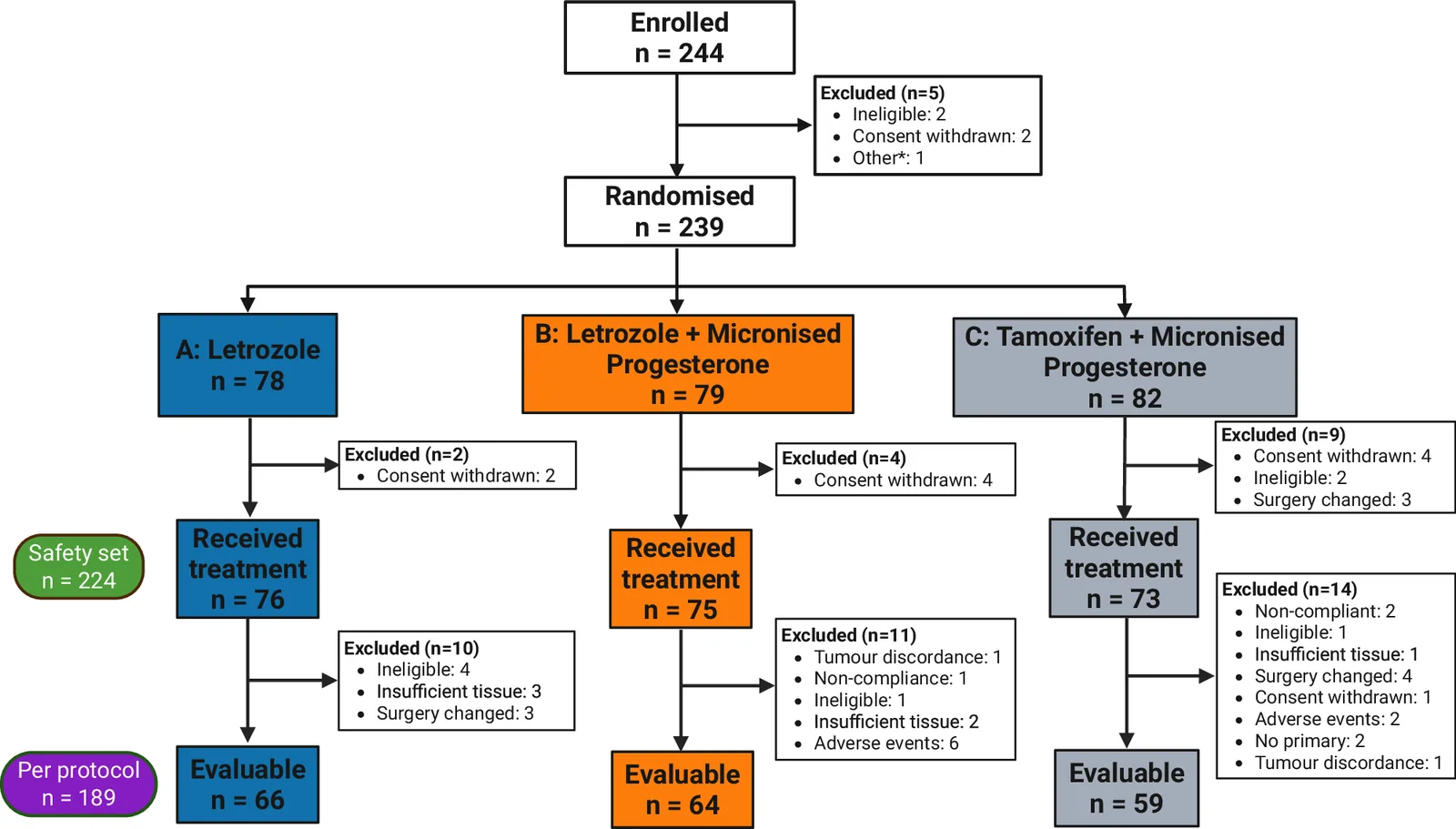
CONSORT diagram. *This patient was excluded due to a site administrative issue which prevented randomisation of the participant. Created in BioRender. Haggstrom, L. (2026) https://BioRender.com/pfryr80.

From February 2018 to June 2024, 250 participants were enrolled across 7 Australian sites. However, all participants at one site (*n* = 6) were excluded due to administrative non-compliance leading to non-adherence with trial protocols, and subsequently, 244 participants remained in the full analysis set. 239 participants were randomised as follows: 78 (32.6%) to letrozole, 79 (33.1%) to letrozole plus MP, and 82 (34.3%) to tamoxifen plus MP. The safety analysis set consisted of 224 participants. After exclusion of 55 participants, a total of 189 evaluable participants, corresponding to 191 tumours (two tumours were included from two patients with multifocal/bilateral tumours), remained in the per protocol population. Compliance data from the evaluable participants is shown in Supplementary Table 2. The reasons for exclusion are illustrated in Fig. 1. Six participants withdrew from the letrozole plus MP arm due to adverse events, two withdrew from tamoxifen plus MP due to adverse events, and none withdrew due to adverse events with letrozole alone.

### Baseline demographics

Baseline demographics in the per protocol population were well balanced between arms as shown in Table 1. The mean age was 68.0 years (standard deviation 8.7 years). The median baseline Ki67 was 10.0% (IQR 5.0–16.3%). There were 148 cases (77.5%) with invasive carcinoma of no special type, 28 (14.7%) of invasive lobular carcinoma, and 15 with other histological subtypes (7.9%). Most tumours were pT1 (*n* = 104, 54.5%), 71 (37.2%) were pT2, and 14 (7.3%) were pT3. No pT4 tumours were included. 69 (36.1%) tumours were node positive and 122 (63.9%) were node negative. Most tumours were highly ER positive (median percent positivity: 95%, IQR 95–95%) and highly PR positive (median percent positivity: 90%, IQR 50–95%).

**Table 1:** Baseline characteristics

Characteristic	Letrozole* *N* = *67*	Letrozole + MP *N* = *64*	Tamoxifen + MP* *N* = *60*
Age, median (Q1, Q3)	68 (61, 73)	68 (62, 72)	68 (61, 75)
pT stage, *n* (%)			
T1a	2 (3%)	2 (3%)	2 (3%)
T1b	8 (12%)	4 (6%)	6 (10%)
T1c	28 (42%)	27 (42%)	28 (47%)
T2	26 (39%)	25 (39%)	19 (32%)
T3	3 (5%)	6 (9%)	5 (8%)
Multifocal or multicentric, *n* (%)	17 (25%)	9 (14%)	13 (22%)
Node positive, *n* (%)	22 (33%)	27 (42%)	20 (33%)
Histological subtype, *n* (%)			
Invasive carcinoma of no special type	50 (75%)	49 (77%)	50 (83%)
Invasive lobular carcinoma	10 (15%)	10 (16%)	8 (13%)
Micropapillary carcinoma	2 (3%)	1 (2%)	0 (0%)
Mucinous carcinoma	3 (5%)	3 (5%)	1 (2%)
Solid papillary carcinoma	1 (2%)	0 (0%)	0 (0%)
Tubular carcinoma	1 (2%)	1 (2%)	0 (0%)
Tubulolobular carcinoma	0 (0%)	0 (0%)	1 (2%)
Baseline grade, *n* (%)			
1	13 (19%)	12 (19%)	13 (22%)
2	39 (58%)	43 (67%)	38 (63%)
3	15 (22%)	9 (14%)	9 (15%)

^*^One participant in the letrozole arm, and another in the tamoxifen plus micronised progesterone arm had two tumours that were included in the per protocol population. The median age is calculated based on the number of participants in each treatment arm, while the other descriptors refer to the number of cancers included in the per protocol population. Percentages may not sum to 100% due to rounding. Q1: quartile 1, Q3: quartile 3.

### Efficacy

**Fig. 2:**
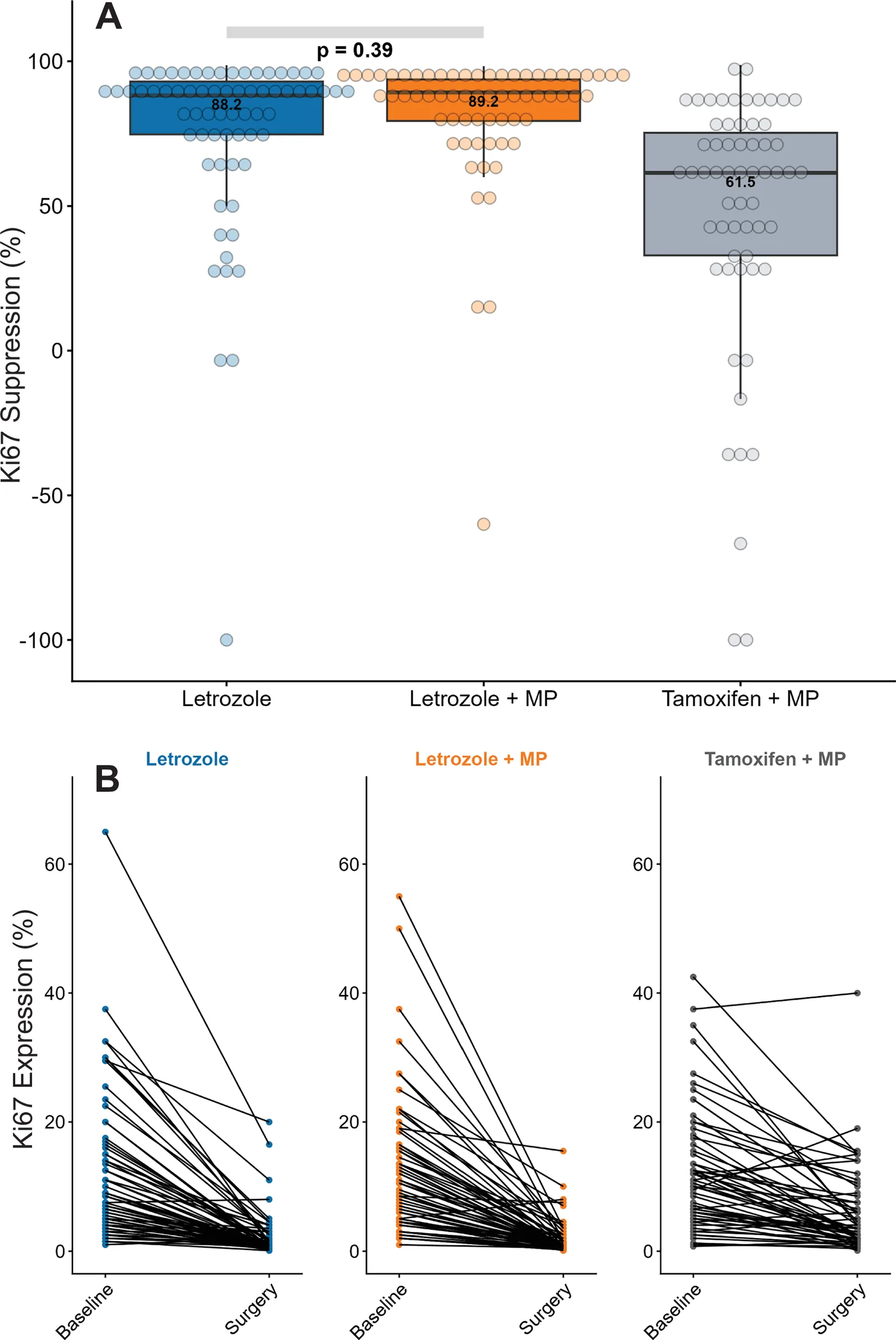
Ki67 Suppression by Treatment Arm. **A** Ki67 suppression (%) in each treatment arm, **B** change in Ki67 expression from baseline to surgery in each treatment arm. MP micronised progesterone.

There was strong correlation between the two anatomical pathologists’ Ki67 results (baseline Ki67 values: Spearman’s rho = 0.960, *p* < 0.001; surgical Ki67 values: Spearman’s rho = 0.931, *p* < 0.001; Supplementary Fig. 1). There was no significant difference in Ki67 suppression between letrozole alone and letrozole plus MP (letrozole: median 88.2%, IQR 74.6–93.0%; letrozole + MP: median: 89.2%, IQR 78.3–93.9%, Wilcoxon test statistic: 1957.5; *p* = 0.39), as shown in Fig. 2. The median Ki67 suppression was numerically lower in the tamoxifen plus MP group (median 61.5%, IQR 32.3–75.9%). Similarly, there was no significant difference in the proportion of patients achieving complete cell cycle arrest (CCCA) with letrozole (*n* = 52, 77.6%) compared to letrozole plus MP (*n* = 53, 82.8%) (χ^2^ = 0.56, *p* = 0.46). A sensitivity analysis using the geometric mean change in Ki67 expression confirmed that there was no significant difference in the change in Ki67 expression between the two letrozole containing groups (student’s *t* test statistic = 0.954, *p* = 0.34) (Supplementary Fig. 2). *Post-hoc* subgroup analyses, as defined by baseline grade, Ki67 expression, PR expression and histology, did not show any differential effect on Ki67 suppression of the addition of MP to letrozole (Supplementary Figs. 4–7). A multivariable linear regression model was developed to predict the log normalised surgical Ki67 expression (F-statistic 40.7; adjusted *R*^2^ 0.385, *p* < 0.001, Supplementary Fig. 8). Two factors remained significant predictors: treatment arm (*p* < 0.001) and baseline log normalised Ki67 values (*p* < 0.001).

### Changes in ER and PR immunohistochemistry

The changes in ER and PR percent positivity are depicted in Table 2. In all treatment groups, the median ER percentage was 95% at both baseline and surgery. There was a significant reduction in PR percentage from a median of 90% at baseline to a median of 20% at surgery with both letrozole (*p* < 0.001) and letrozole plus MP (*p* < 0.001), but not with tamoxifen plus MP (*p* = 0.5). There was no difference in the median reduction in the ER Allred score with letrozole (median 0%, IQR: 0–0%) versus letrozole plus MP (median 0%, IQR: 0-0%) (W = 2092, *p* = 0.72). There was also no significant difference in the median reduction in the PR Allred score with letrozole (33.3%, IQR: 12.5–57.1%) versus letrozole plus MP (median 37.5%, IQR: 12.5–57.1%) (W = 1954.5, *p* = 0.38). There was a weak negative correlation between ER Allred score suppression and Ki67 suppression (Spearman’s rho = −0.16, *p* = 0.02) and a moderate positive correlation between Ki67 suppression and PR Allred Score suppression (Spearman’s rho = 0.35, *p* < 0.001; Supplementary Fig. 9).

**Table 2:** Changes in Ki67, ER and PR

	Baseline			Surgery		
Treatment	Let *N* = *67*	Let + MP *N* = *64*	Tam + MP *N* = *60*	Let *N* = *67*	Let + MP *N* = *64*	Tam + MP *N* = *60*
Ki67 % suppression, median (Q1, Q3)	–	–	–	88.2 (74.6, 93.0)	89.2 (78.3, 93.9)	61.5 (32.3, 75.9)
Ki67 %, median (Q1, Q3)	8.5 (4.5, 16.0)	10.5 (5.0, 16.3)	10.8 (6.0, 17.0)	1.0 (0.5, 2.5)	1.0 (0.5, 2.0)	3.5 (2.0, 7.5)
ER %, median (Q1, Q3)	95 (95, 95)	95 (95, 95)	95 (95, 95)	95 (95, 95)	95 (95, 95)	95 (95, 95)
PR %, median (Q1, Q3)	90 (50, 95)	90 (35, 95)	90 (50, 95)	20* (4, 70)	20* (2, 70)	90 (33, 95)
ER Allred score, *n* (%)						
High	67 (100%)	64 (100%)	59 (98%)	63 (94%)	64 (100%)	58 (97%)
Intermediate	0 (0%)	0 (0%)	1 (2%)	4 (6%)	0 (0%)	2 (3%)
Zero-low	0 (0%)	0 (0%)	0 (0%)	0 (0%)	0 (0%)	0 (0%)
PR Allred score, *n* (%)						
High	47 (70%)	46 (72%)	44 (73%)	15 (22%)	15 (23%)	39 (65%)
Intermediate	17 (25%)	14 (22%)	13 (22%)	29 (43%)	24 (38%)	19 (32%)
Zero-low	3 (4.5%)	4 (6.3%)	3 (5.0%)	23 (34%)	25 (39%)	2 (3.3%)

Baseline ER and PR Allred scores were grouped into three groups as follows: zero-low if Allred score was 0–3, intermediate if Allred score was 4-6, and high if Allred score was 8.

*ER* oestrogen receptor *PR* progesterone receptor, *MP* micronised progesterone, *Q1* quartile 1, *Q3* quartile 3.

*p value < 0.01.

### Safety

All adverse reactions that were deemed possibly, probably, or definitely related to therapy with an incidence of ≥1% across the entire cohort are shown in Table 3. Only two adverse reactions (hot flushes and fatigue) had an incidence of >10% in the entire cohort. Grade 3 and above reactions were uncommon, with an incidence of 3/224 (1.3%) in the cohort. Fatigue appeared more frequent with tamoxifen plus MP arm (35.6%), compared to letrozole (17.1%) or letrozole plus MP (22.7%). Hot flushes appeared less frequent with letrozole plus MP (13.3%) compared to letrozole (22.4%) or tamoxifen plus MP (20.5%). Dizziness was more common in the MP-containing arms. The incidence and relative risk of each adverse reaction are shown in Supplementary Figs. 12 and 13.

**Table 3:** All adverse reactions with incidence of ≥1% and all grade 3+ adverse reactions

Adverse reaction, *n* (%)	Letrozole *(n* = *76)*			Letrozole + MP *(n* = *75)*			Tamoxifen + MP *(n* = *73)*		
Grade	**G1**	**G2**	**G3**+	**G1**	**G2**	**G3**+	**G1**	**G2**	**G3**+
Anorexia	1 (1.3)	0	0	4 (5.3)	0	0	0	0	0
Anxiety	2 (2.6)	0	0	1 (1.3)	0	0	0	0	0
Arthralgia	6 (7.9)	0	0	3 (4.0)	0	0	0	1 (1.4)	0
Constipation	2 (2.6)	0	0	1 (1.3)	0	0	1 (1.4)	0	0
Diarrhoea	5 (6.6)	0	0	5 (6.7)	1 (1.3)	0	0	0	0
Dizziness	1 (1.3)	0	0	7 (9.3)	2 (2.7)	1 (1.3)	6 (8.2)	0	0
Dysgeusia	2 (2.6)	0	0	1 (1.3)	0	0	1 (1.4)	0	0
Fatigue	13 (17.1)	0	0	14 (18.7)	3 (4.0)	0	22 (30.1)	4 (5.5)	0
Gastroesophageal reflux disease	1 (1.3)	0	0	2 (2.7)	0	0	0	0	0
Headache	5 (6.6)	0	0	5 (6.7)	0	0	4 (5.5)	1 (1.4)	0
Hot flushes	15 (19.7)	2 (2.6)	0	8 (10.7)	2 (2.7)	0	13 (17.8)	2 (2.7)	0
Insomnia	3 (3.9)	0	0	3 (4.0)	1 (1.3)	0	4 (5.5)	0	0
Irritability	1 (1.3)	0	0	0	0	0	2 (2.7)	0	0
Lethargy	0	0	0	0	2 (2.7)	0	1 (1.4)	0	0
Myalgia	2 (2.6)	0	0	4 (5.3)	0	0	0	0	0
Nausea	6 (7.9)	0	0	6 (8.0)	1 (1.3)	0	3 (4.1)	1 (1.4)	0
Takotsubo cardiomyopathy	0	0	0	0	0	0	0	0	1 (1.4)
Vaginal inflammation	0	0	0	0	0	0	2 (2.7)	0	1 (1.4)

Adverse reactions included were those deemed to be possibly, probably or definitely related to study treatment by the investigator.

*MP* micronised progesterone.

Seven participants experienced serious adverse events, summarised in Supplementary Table 7. Three of these events were deemed to be possibly, probably or definitely related to the trial therapy by the local investigator, including two transient ischemic attacks with letrozole plus MP, and Takotsubo cardiomyopathy occurring in the postoperative period in a patient treated with tamoxifen plus MP. Eight participants withdrew from the trial due to adverse events: six from letrozole plus MP and two from tamoxifen plus MP (Supplementary Table 8). Two withdrawals were deemed by the investigators to be unrelated to treatment, and the remainder were considered possibly or probably treatment related.

## Discussion

To our knowledge, the WinPro trial is the first randomised trial evaluating the addition of MP to letrozole in early-stage hormone receptor positive breast cancer. There was no difference in Ki67 suppression with the addition of MP to letrozole compared to letrozole alone in the per protocol population or in *post-hoc* subgroup analyses. The addition of MP to letrozole appeared to reduce hot flushes but increase low-grade dizziness. Treatment discontinuation due to adverse events appeared more frequent in the two MP-containing groups compared to letrozole alone. We observed that Ki67 suppression was correlated with a reduction in PR Allred Score, consistent with effective inhibition of classical ER signalling after endocrine therapy. These findings suggest that at the dose used in the WinPro trial, the use of MP may be a helpful adjunct for managing hot flushes due to aromatase inhibitors in select cases, without conferring pro-tumorigenic effects; however, studies in the adjuvant setting with longer-term intervention and follow-up are needed to confirm this.

There are several potential explanations for the negative result of the primary endpoint. First, the median Ki67 suppression was 88.2% with letrozole alone compared to 89.2% with letrozole plus MP. This degree of Ki67 suppression with letrozole was higher than reported in the literature and is likely a consequence of our inclusion criteria, which, by excluding ER low or PR negative tumours, led to preferential inclusion of tumours with good biology, luminal A characteristics and low baseline Ki67. In the hormone receptor positive, HER2 negative subgroup of the POETIC trial treated with letrozole, the median baseline Ki67 was 14.5%, and after 2 weeks was 2.8%, with median Ki67 suppression of 76.8%^7^. In the WinPro trial, the selection of a highly endocrine-sensitive cohort and unexpected overperformance of the control arm may have reduced the dynamic range and statistical power to detect small differences in Ki67 suppression between the two letrozole-containing groups. Indeed, the median post-treatment Ki67 was only 1.0% in both letrozole-containing arms, suggesting a potential floor effect. Second, the dose of MP used in this study was based on the dose that is licensed for use in menopausal hormone therapy^14–17^, and it is possible that a higher dose or longer treatment duration is required for therapeutic effects. The Ki67 suppression with tamoxifen plus MP appeared lower than the other two arms (61.5%), which is largely consistent with literature, where the Ki67 suppression with short-term tamoxifen alone has ranged from 40 to 59.5%^18–20^.

Overall, we observed a low frequency of adverse reactions as expected due to the short-term administration of preoperative endocrine therapy in this trial. While letrozole plus MP appeared to reduce hot flushes compared to letrozole alone, this was countered by an increase in dizziness, a known side effect of MP, limiting the potential utility of MP as an intervention to manage hot flushes. Nocte administration of MP is known to reduce the incidence of dizziness but was not mandated in the trial protocol. There was also an increase in treatment discontinuations and serious adverse events, including transient ischemic attacks in the MP arms. Both transient ischemic attacks occurred in elderly patients above 75 years of age and were deemed possibly related to the interventional product by the local investigator. Although causality cannot be established from these isolated events, their occurrence underscores the importance of ongoing safety monitoring in future studies evaluating longer durations of MP exposure.

Controversy regarding the role of progestogens in breast cancer exists, as while progestins were historically used to treat breast cancer with responses superior to tamoxifen^9^, the Women’s Health Initiative showed that synthetic progestins increased the incidence of breast cancer^21^; however, the findings of this have been heavily criticised^22–24^. Preclinical work has demonstrated that progesterone inhibits growth of established ER+ breast cancer via reprogramming where ER binds to chromatin^8^. The PIONEER trial was a window of opportunity study where postmenopausal women with ER + , HER2- breast cancer were randomised to letrozole alone or with the progestin megestrol acetate^11^. Two doses of megestrol acetate were used: a high dose, which was used historically to treat advanced breast cancer^25^, and a lower dose approved for treating hot flushes^26^. They demonstrated a greater reduction in Ki67 with the addition of megestrol acetate to letrozole (80% with both megestrol doses combined vs 71% with letrozole, *p* = 0.013), and that the effects of megestrol acetate were similar regardless of the dose used^11^. Furthermore, using paired ERα chromatin immunoprecipitation sequencing, the authors observed a reduction in ER binding to DNA after administration of letrozole, and an even greater reduction in binding in the letrozole plus megestrol acetate group, consistent with the model that PR reprograms ER binding to chromatin^8,11^. There has been concern that progesterone, when administered in the context of oestrogen deprivation similar to that induced by aromatase inhibition, could have detrimental effects^27,28^. However, the data from the WinPro trial provide evidence that at the administered schedule, PR agonism with MP is not pro-tumorigenic. Although MP and megestrol acetate are both PR agonists, they differ substantially in pharmacology. Megestrol acetate has additional activity at the androgen and glucocorticoid receptors, whereas MP is a relatively selective PR ligand^29–31^. Therefore, the greater Ki67 suppression observed in PIONEER may reflect receptor crosstalk beyond PR activation alone. Such effects may, in part, relate to androgen receptor signalling, which has established anti-proliferative activity in ER-positive breast cancer^29,30^.

The WinPro trial has several limitations. First, there was no tamoxifen-alone control arm, meaning that the tamoxifen plus MP arm was considered as purely exploratory and it is not possible to determine whether the addition of MP to tamoxifen had neutral, positive or detrimental effects compared to tamoxifen alone. Second, this trial provides no data on the long-term safety or efficacy of MP, a limitation intrinsic to short-term window of opportunity studies, although the post-treatment Ki67 is accepted to be a valid surrogate for long-term outcomes due to its robust prognostic association^7^. Furthermore, the analysis of Ki67 has several potential limitations, due to heterogeneous expression or sampling bias, and interobserver variability^32^. For instance, there is likely to be greater variability in Ki67 staining in surgical specimens than biopsy samples, and central pathology review did not attempt to match tumour-rich regions of surgical specimens to the paired biopsy samples. The potential sampling bias due to tumour heterogeneity was minimised through the use of central Ki67 staining, and through blinded independent review by two independent pathologists. Additionally, most patients had high baseline ER expression, so the results may not be generalisable to patients with lower baseline ER values. There was also a slightly higher drop-out rate in the tamoxifen plus MP arm compared to the other two groups; however, as most of the reasons for attrition in this group were due to changes in the surgery date, this is unlikely to bias the results. Finally, as previously discussed, the dose of MP used in this trial may have been a suboptimal therapeutic dose. Further analysis of spatial transcriptomics data from the trial is underway and will provide evidence regarding whether engagement of PR was sufficient by MP.

In conclusion, there was no difference in Ki67 suppression with letrozole plus MP compared to letrozole alone, and the addition of MP appeared to reduce hot flushes. These findings provide short-term evidence suggesting that MP at the dose administered in the WinPro trial does not promote proliferation in ER+ breast cancer.

## Methods

### Participants

Eligible participants were postmenopausal women with early-stage ER+, PR+, HER2- breast cancer. ER and PR positivity was defined as ≥10% of cells staining positive based on local pathological assessment. Tumour size was ≥5 mm as determined by clinical assessment or imaging. Participants were at least 18 years old and were able to understand all patient information and to provide written informed consent to participate in the trial. Participants were excluded if they were currently receiving hormone therapies, had locally advanced and inoperable or inflammatory breast cancer, clinical evidence of metastatic disease, were treated with other preoperative systemic therapy, nut allergy, or a prior history of endometrial cancer or venous thromboembolism.

### Study design and treatment

Participants were recruited from 7 Australian hospitals. Participants were randomised 1:1:1 using computer-generated randomisation to letrozole 2.5 mg PO daily, letrozole 2.5 mg and MP 300 mg PO daily, or tamoxifen 20 mg PO daily and MP 300 mg PO daily. Neither the patient nor the treating physician was blinded to the treatment allocation. After randomisation, patients were treated with 14 days (±3 days) of preoperative therapy, with the last dose of medication administered on the day prior to their surgery.

Slides were stained in a central diagnostic laboratory for ER, PR and Ki67. Ki67 was scored independently by two experienced breast anatomical pathologists (KM, AP) who were blinded to treatment allocation and the mean of both pathologists’ Ki67 results was used for analysis of the primary endpoint. One pathologist reported ER and PR, and the second pathologist reviewed the first pathologist’s ER/PR results for concordance. Antibodies used and methodology are described in Supplementary Table 1. Participants received adjuvant therapy as per standard of care recommended by their treating physicians.

The primary endpoint was the percent proportional reduction (“Ki67 suppression”) in tumoural Ki67 defined as the difference between the post-treatment and pre-treatment Ki67 (%) divided by the pre-treatment Ki67 (%). Secondary endpoints were safety and tolerability defined using the National Cancer Institute Common Terminology Criteria for Adverse Events version 4.0 (NCI-CTCAE v4.0). Translational endpoints were changes in ER and PR protein expression, and spatial transcriptomics analyses which will be reported separately. No long-term follow-up was conducted.

### Statistical analysis

Statistical analyses were conducted in SAS and RStudio (version 4.3.3). Baseline demographics were analysed descriptively. The distribution of the Ki67 suppression was inspected and tested using the Shapiro–Wilk test (*p* < 0.001), and due to non-normal distribution, a two-sided Wilcoxon Rank Sum test was used. We allocated 5% alpha to the comparison between the two letrozole containing arms, and considered the tamoxifen plus MP arm as exploratory. Previous results from the IMPACT trial observed a reduction in the geometric mean of Ki67 after 2 weeks of tamoxifen of 59.5% and with anastrozole of 76%^33^. A power calculation determined that a sample size of 200 would be needed to detect an improvement in Ki67 suppression from 76% with letrozole alone to 92% in the letrozole plus MP arm, allowing for 4% dropouts, with 80% power and a *p*-value of 0.05. Descriptive *post-hoc* subgroup analyses investigating Ki67 suppression in groups defined by baseline grade, Ki67 expression, PR expression and histology were also conducted and are considered purely exploratory.

Other endpoints included the proportion of tumours achieving CCCA, which is defined as a post-treatment Ki67 of ≤2.7%^34^. The Chi-squared test was used to compare the proportion of patients achieving CCCA between the two letrozole containing arms, with the tamoxifen plus MP arm again being considered as exploratory. A sensitivity analysis was also conducted to assess the change in geometric mean between the two letrozole containing groups. The log of the proportional change in Ki67 between timepoints was calculated for each patient as follows: *log*_*e*_*(surgical Ki67/ baseline Ki67)*, consistent with the approach of Hurvitz and colleagues^35^. The distribution of this proportional change was assessed and, given normality of the log-transformed data and homogeneity of variance was confirmed (Bartlett’s test, *p* = 0.31), a t-test was used to compare the log- normalised proportional Ki67 change between the two letrozole containing groups. Changes in ER and PR percentages and Allred scores were evaluated using two-tailed Wilcoxon signed rank tests as the data were not normally distributed. Correlations between changes in ER and PR expression, and Ki67 suppression were evaluated using Spearman’s rho.

A linear mixed effects model was also constructed to determine whether baseline clinicopathological factors could predict the surgical Ki67 expression. Ki67 values were log normalised for use in the model. Included predictors were treatment, histological subtype, baseline Ki67 values, grade, baseline ER Allred score, baseline PR Allred score, tumour size, treating hospital (treated as a random effect) and nodal positivity. Model performance was evaluated using both backwards and forwards stepwise elimination procedures, and inspection of Akaike Information Criterion values. Both methods of elimination converged on the final model, which included the baseline Ki67 and treatment as predictors.

### Trial oversight

The study was designed by academic investigators and was registered on 01/06/2018 in the Australian New Zealand Trials Clinical Trials Registry (registration number: ACTRN12618000928213). Besins Healthcare provided MP for the study and had no role in the design or conduct of the trial. The study received central ethical approval from St Vincent’s Hospital Human Research Ethics Committee (2019/ETH03548, HREC/17/SVH/255) and local ethical and governance approval at the following sites: South Western Sydney Local Health District Human Research Ethics Committee (HREC), Chris O’Brien Lifehouse, Nepean Blue Mountains Local Health District HREC, The Mater Hospital Sydney, Peter MacCallum Cancer Centre HREC, and Central Adelaide Local Health Network HREC). St Vincent’s Hospital HREC (EC00140) has been accredited by NSW Ministry of Health as a Lead HREC under the model for single ethical and scientific review and certified by the NHMRC under the National Certification Scheme. This lead HREC is constituted and operates in accordance with the National Health and Medical Research Council’s *National Statement on Ethical Conduct in Human Research* and the

CPMP/ICH Note for Guidance on Good Clinical Practice. No HREC members with a conflict of interest were present for review of this project. All authors confirm that the study was conducted in accordance with the study protocol and principles of International Conference of Harmonisation Good Clinical Practices and the National Statement on Ethical Conduct in Human Research. The trial protocol and statistical analysis plan can be accessed in the Supplementary Information. The public and patients were not involved in the design, conduct or reporting of the trial.

## Supplementary information

Supplementary Information

Supplementary Materials

## Data Availability

Owing to ethical restrictions, raw data from the trial has not been made externally available. The corresponding author can be contacted for reasonable requests to access data.

## Code availability

Code for analysis is available at https://github.com/swarbricklab/winpro_paper.

## References

[1] The Cancer Genome Atlas Network. Comprehensive molecular portraits of human breast tumours. *Nature* **490**, 61–70 (2012). 10.1038/nature11412 PMC3465532 23000897

[2] Pan, H. et al. 20-year risks of breast-cancer recurrence after stopping endocrine therapy at 5 years. *N. Engl. J. Med.* **377**, 1836–1846 (2017). 10.1056/NEJMoa1701830 PMC5734609 29117498

[3] Wei, J. et al. Beyond the 5-year mark: adherence to and continuation of extended adjuvant endocrine therapy in non-metastatic breast cancer patients. *J. Clin. Oncol.* **43**, 523–523 (2025). 10.1007/s10549-025-07811-6 40866758

[4] Collin, L. J. et al. Early discontinuation of endocrine therapy and recurrence of breast cancer among premenopausal women. *Clin. Cancer Res.* **27**, 1421–1428 (2021). 10.1158/1078-0432.CCR-20-3974 PMC7925421 33334905

[5] Murphy, C. C., Bartholomew, L. K., Carpentier, M. Y., Bluethmann, S. M. & Vernon, S. W. Adherence to adjuvant hormonal therapy among breast cancer survivors in clinical practice: a systematic review. *Breast Cancer Res. Treat.* **134**, 459–478 (2012). 10.1007/s10549-012-2114-5 PMC3607286 22689091

[6] Ziller, V. et al. Adherence to adjuvant endocrine therapy in postmenopausal women with breast cancer. *Ann. Oncol.* **20**, 431–436 (2009). 10.1093/annonc/mdn646 19150950

[7] Smith, I. et al. Long-term outcome and prognostic value of Ki67 after perioperative endocrine therapy in postmenopausal women with hormone-sensitive early breast cancer (POETIC): an open-label, multicentre, parallel-group, randomised, phase 3 trial. *Lancet Oncol.* **21**, 1443–1454 (2020). 10.1016/S1470-2045(20)30458-7 PMC7606901 33152284

[8] Mohammed, H. et al. Progesterone receptor modulates ERα action in breast cancer. *Nature* **523**, 313–317 (2015). 10.1038/nature14583 PMC4650274 26153859

[9] Muss, H. B. et al. Tamoxifen versus high-dose oral medroxyprogesterone acetate as initial endocrine therapy for patients with metastatic breast cancer: a Piedmont Oncology Association study. *J. Clin. Oncol.* **12**, 1630–1638 (1994). 10.1200/JCO.1994.12.8.1630 8040675

[10] Ingle, J. N. et al. Randomized clinical trial of megestrol acetate versus tamoxifen in paramenopausal or castrated women with advanced breast cancer. *Am. J. Clin. Oncol.* **5**, 155–160 (1982). 10.1097/00000421-198204000-00062 7046412

[11] Burrell, R. A. et al. Evaluating progesterone receptor agonist megestrol plus letrozole for women with early-stage estrogen-receptor-positive breast cancer: the window-of-opportunity, randomized, phase 2b, PIONEER trial. *Nat. Cancer*10.1038/s43018-025-01087-x (2026). PMC12858400 41492093

[12] United States Food and Drug Administration. Drug Approval Package: Prometrium (Progesterone) Capsules, https://www.accessdata.fda.gov/drugsatfda_docs/nda/98/020843_s000_PrometriumTOC.cfm (2005).

[13] Fitzpatrick, L. A. & Good, A. Micronized progesterone: clinical indications and comparison with current treatments. *Fertil. Steril.* **72**, 389–397 (1999). 10.1016/s0015-0282(99)00272-1 10519605

[14] Judd, H. L. et al. Effects of hormone replacement therapy on endometrial histology in postmenopausal women: The Postmenopausal Estrogen/Progestin Interventions (PEPI) trial. *JAMA* **275**, 370–375 (1996). 10.1001/jama.1996.03530290040035 8569016

[15] Cuadros, J. L. et al. Metabolic and hormonal parameters in post-menopausal women 10 years after transdermal oestradiol treatment, alone or combined to micronized oral progesterone. *Gynecol. Endocrinol.* **27**, 156–162 (2011). 10.3109/09513590.2010.487613 20500105

[16] Auerbach, P., Zipfel, L., Yang, H. M., Allgood, A. & Guo, C. Y. Evaluation of safety and efficacy of micronized progesterone in induction of secretory conversion of endometrium and withdrawal bleeding in women with secondary amenorrhea. *Fertil. Steril.* **94**, S188–S189 (2010).

[17] Hitchcock, C. L. & Prior, J. C. Oral micronized progesterone for vasomotor symptoms-a placebo-controlled randomized trial in healthy postmenopausal women. *Menopause* **19**, 886–893 (2012). 10.1097/gme.0b013e318247f07a 22453200

[18] Cohen, A. L. et al. POWERPIINC (PreOperative Window of Endocrine TheRapy Provides Information to Increase Compliance) trial: changes in tumor proliferation index and quality of life with 7 days of preoperative tamoxifen. *Breast* **31**, 219–223 (2017). 10.1016/j.breast.2016.11.016 27915204

[19] Llombart Cussac, A. et al. 294MO Preoperative window-of-opportunity study with giredestrant or tamoxifen (tam) in premenopausal women with estrogen receptor-positive (ER+)/human epidermal growth factor receptor 2-negative (HER2-) and Ki67 ≥10% early breast cancer (EBC): The EMPRESS study. *Ann. Oncol.* **36**, S323 (2025).

[20] Dowsett, M. et al. Short-term changes in Ki-67 during neoadjuvant treatment of primary breast cancer with anastrozole or tamoxifen alone or combined correlate with recurrence-free survival. *Clin. Cancer Res.* **11**, 951s–958s (2005). 15701892

[21] Chlebowski, R. T. et al. Estrogen plus progestin and breast cancer incidence and mortality in postmenopausal women. *JAMA* **304**, 1684–1692 (2010). 10.1001/jama.2010.1500 PMC5142300 20959578

[22] Bluming, A. Z. Introduction: estrogen reconsidered: exploring the evidence for estrogen’s benefits and risks. *Cancer J.* **28**, 157–162 (2022). 10.1097/PPO.0000000000000602 35594461

[23] Langer, R. D., Hodis, H. N., Lobo, R. A. & Allison, M. A. Hormone replacement therapy - where are we now? *Climacteric* **24**, 3–10 (2021). 10.1080/13697137.2020.1851183 33403881

[24] Tan, D. A. & Dayu, A. R. B. Menopausal hormone therapy: why we should no longer be afraid of the breast cancer risk. *Climacteric* **25**, 362–368 (2022). 10.1080/13697137.2022.2035711 35147073

[25] Abrams, J. et al. Dose-response trial of megestrol acetate in advanced breast cancer: cancer and leukemia group B phase III study 8741. *J. Clin. Oncol.* **17**, 64–73 (1999). 10.1200/JCO.1999.17.1.64 10458219

[26] Loprinzi, C. L. et al. Megestrol acetate for the prevention of hot flashes. *N. Engl. J. Med.* **331**, 347–352 (1994). 10.1056/NEJM199408113310602 8028614

[27] Horwitz, K. B. & Sartorius, C. A. 90 years of progesterone: progesterone and progesterone receptors in breast cancer: past, present, future. *J. Mol. Endocrinol.* **65**, T49–T63 (2020). 10.1530/JME-20-0104 PMC8525510 32485679

[28] Tanos, T. et al. Progesterone/RANKL is a major regulatory axis in the human breast. *Sci. Transl. Med.* **5**, 182ra155–182ra155 (2013). 10.1126/scitranslmed.3005654 23616122

[29] Moore, N. L., Hickey, T. E., Butler, L. M. & Tilley, W. D. Multiple nuclear receptor signaling pathways mediate the actions of synthetic progestins in target cells. *Mol. Cell Endocrinol.* **357**, 60–70 (2012). 10.1016/j.mce.2011.09.019 21945474

[30] Hickey, T. E. et al. The androgen receptor is a tumor suppressor in estrogen receptor-positive breast cancer. *Nat. Med.* **27**, 310–320 (2021). 10.1038/s41591-020-01168-7 33462444

[31] Manivannan, M. et al. Activated glucocorticoid receptor is an estrogen receptor silencer in ER+ metastatic breast cancer. *EMBO Mol. Med.* 10.1038/s44321-025-00342-z (2025). PMC12808765 41261233

[32] Nielsen, T. O. et al. Assessment of Ki67 in Breast Cancer: updated Recommendations From the International Ki67 in Breast Cancer Working Group. *J. Natl. Cancer Inst.* **113**, 808–819 (2021). 10.1093/jnci/djaa201 PMC8487652 33369635

[33] Dowsett, M. et al. Prognostic value of Ki67 expression after short-term presurgical endocrine therapy for primary breast cancer. *J. Natl. Cancer Inst.* **99**, 167–170 (2007). 10.1093/jnci/djk020 17228000

[34] Giltnane, J. M. et al. Genomic profiling of ER(+) breast cancers after short-term estrogen suppression reveals alterations associated with endocrine resistance. *Sci. Transl. Med.* **9**, 10.1126/scitranslmed.aai7993 (2017). PMC5723145 28794284

[35] Hurvitz, S. A. et al. Neoadjuvant palbociclib plus either giredestrant or anastrozole in oestrogen receptor-positive, HER2-negative, early breast cancer (coopERA Breast Cancer): an open-label, randomised, controlled, phase 2 study. *Lancet Oncol.* **24**, 1029–1041 (2023). 10.1016/S1470-2045(23)00268-1 37657462

