# Supplementary material for: The WinPro trial: window-of-opportunity study of endocrine therapy with micronised progesterone in ER-positive breast cancer
Source: NPJ Breast Cancer. 2026 Jul 21;12:144. doi: 10.1038/s41523-026-01008-w (PMC13752282; doi:10.1038/s41523-026-01008-w)
Supplement: Supplementary file 1 [file 41523_2026_1008_MOESM1_ESM.pdf]

Supplementary Information

The WinPro Trial: Window-of-Opportunity Study of Endocrine Therapy with Micronised Progesterone in ER-Positive Breast Cancer.

Supplementary Table 1: Immunohistochemistry Methods

Protein	Antigen Retrieval Conditions	Clone	Source	Host species
Ki67 (MIB1)	Retrieval: CC1, 36min, 95C<br>Incubation: 28min, 36C<br>UltraView Detection	30-9	Roche Ventana (790-4286)	Rabbit
ERα	Retrieval: CC1, 64min, 95C<br>Incubation: 32min, 36C<br>UltraView Detection	SP1	Roche Ventana (790-4324)	Rabbit
PR	Retrieval: CC1, 64min, 95C<br>Incubation: 16min, 36C<br>UltraView detection	1E2	Roche Ventana (790-2223)	Rabbit

Supplementary Table 2: Treatment Compliance

Dose Received		Letrozole *N = 67*	Letrozole + MP *N = 64*	Tamoxifen + MP *N = 60*
Letrozole, n (%)	0 mg	0	0	60 (100%)
	27.5 mg	1 (1.5%)	0	0
	30 mg	2 (3.0%)	0	0
	32.5 mg	1 (1.5%)	2 (3.1%)	0
	35 mg	62 (92.5%)	62 (96.9%)	0
Micronised progesterone, n (%)	0 mg	67 (100%)	0	0
	3600 mg	0	0	2 (3.4%)
	3900 mg	0	3 (4.7%)	1 (1.7%)
	4100 mg	0	0 (0%)	2 (3.4%)
	4200 mg	0	61 (95%)	53 (90%)
	4800 mg	0	0 (0%)	1 (1.7%)
Tamoxifen, n (%)	0 mg	67 (100%)	64 (100%)	0
	240 mg	0	0	2 (3.4%)
	260 mg	0	0	2 (3.4%)
	280 mg	0	0	54 (90.0%)
	320 mg	0	0	1 (1.7%)

Note: 14 days of treatment with letrozole, MP, and tamoxifen equates to 35 mg, 4200 mg and 280 mg, respectively. MP: micronised progesterone.

Supplementary Table 3: Complete Cell Cycle Arrest

**Complete Cell Cycle Arrest**	**Letrozole *N* = 67**	**Letrozole + MP *N* = 64**	**Tamoxifen + MP *N* = 60**	**Total *N* = 191**
Yes	52 (77.6%)	53 (82.8%)	28 (46.7%)	133 (69.6%)
No	15 (22.4%)	11 (17.2%)	32 (53.3%)	58 (30.4%)

Note: Complete cell cycle arrest is defined as a post-treatment Ki67 of ≤2.7%. MP: micronised progesterone.

Supplementary Table 4: Multiple Linear Regression Model

	Dependent variable:	*P value*
	Log transformed surgical Ki67	
Treatment⁺: letrozole + micronised progesterone	-0.131 (0.168)	0.44
Treatment⁺: tamoxifen + micronised progesterone	1.063 (0.171)	<0.001*
Log transformed baseline Ki67	0.636 (0.081)	<0.001*
Constant	-1.248 (0.208)	<0.001*
Observations	191	
R^2	0.395	
Adjusted R^2	0.385	
Residual standard error	0.959 (df = 187)	
F statistic	40.719^{***} (df = 3; 187)	

Notes: results presented as estimate (standard error) unless otherwise stated, ⁺: letrozole treatment group considered as reference; df: degrees of freedom; *p<0.001.

Supplementary Table 5: Ki67 Suppression in Post-Hoc Subgroups

**Baseline Characteristic**	**Ki67 Suppression, median (Q1, Q3)**

Treatment	Letrozole<br>N = 67	Letrozole + Prometrium<br>N = 64	Tamoxifen + Prometrium<br>N = 60
**Baseline PR Status**			
Zero-low (n = 10)	91.4 (90.2, 91.7)	87.6 (74.1, 95.5)	84.0 (61.5, 88.6)
Intermediate (n = 44)	87.5 (66.2, 92.0)	86.6 (76.5, 89.2)	50.0 (33.3, 74.5)
High (n = 137)	86.1 (74.6, 93.3)	90.0 (80.0, 94.3)	62.6 (30.7, 75.6)
**Baseline Grade**			
1 (n = 38)	91.7 (77.8, 93.0)	88.9 (67.9, 92.4)	64.3 (60.0, 72.2)
2 (n = 120)	86.1 (63.6, 91.7)	88.4 (80.0, 94.3)	51.0 (30.0, 74.5)
3 (n = 33)	90.2 (74.6, 97.3)	92.6 (89.1, 93.6)	81.5 (64.7, 86.2)
**Baseline Ki67**			
Low (n = 95)	85.4 (50.0, 91.7)	87.7 (75.0, 91.4)	60.8 (25.0, 70.0)
High (n = 96)	90.6 (80.0, 95.5)	92.0 (81.8, 96.2)	64.9 (40.4, 83.3)
**Histology**			
Invasive breast carcinoma of no special type (n=149)	87.5 (75.0, 91.7)	89.2 (80.0, 93.3)	62.6 (40.0, 75.0)
Invasive lobular carcinoma (n=28)	91.1 (77.8, 93.3)	91.6 (76.5, 97.0)	38.5 (-83.4, 84.4)
Other (n=14)	91.7 (30.0, 98.0)	86.0 (84.6, 92.6)	60.0 (50.0, 70.0)

Notes: Baseline PR status was grouped into three groups based on the Allred score as follows: zero-low if Allred score was 0-3, intermediate if Allred score was 4-6, and high if Allred score was 7-8. Baseline Ki67 was split into two groups using a cutoff of ≥10% as high and <10% as low.

Supplementary Table 6: Summary of Adverse Events in Safety Analysis Set

Event	Letrozole *(n=76)*	Letrozole + MP *(n=75)*	Tamoxifen + MP *(n=73)*
All AEs	170	166	182
All ARs	85	88	89
Grade 3+ ARs	4	8	12
SAEs	0	0	4
SARs	0	2	1
AEs leading to treatment discontinuation	0	6	2
ARs leading to treatment discontinuation	0	5	1
Grade 5 AEs	0	0	1

Abbreviations: AE: adverse event; AR: adverse reaction; MP: micronised progesterone; SAE: serious adverse event; SAR: serious adverse reaction.

Supplementary Table 7: Serious Adverse Events and Reactions

SAE type	Age at enrolment	Treatment	Days of treatment completed	Severity	Relatedness	Summary	Outcome
SAR	76	LMP	12	Mild	Possible	TIA. Pre-existing vascular risk factors.	Resolved
SAE	80	TMP	14	Death	Unrelated	Patient hospitalized 12 days post-operatively with AKI on CKD, T2RF, pneumonia, CCF, anaemia. VQ scan negative for PE.	Death
SAR	86	LMP	3	Severe	Possible	TIA.	Resolved
SAE	64	TMP	14	Not reported	Unrelated	New dx of pancreatic cancer post-operatively requiring hospitalization for ERCP and stent insertion for obstructive jaundice.	Resolved
SAR	71	TMP	14	Life-threatening	Possible	Takotsubo's cardiomyopathy occurred 5 days post-operatively.	Resolved
SAE	62	TMP	7	Severe	Unrelated	AKI, diarrhea, nausea, vaginal inflammation, blurred vision, acute infection, haematuria, maculo-papular rash.	Resolved
SAE	61	TMP	14	Severe	Unrelated	Patient presented to hospital with wound infection, treated with oral antibiotics.	Resolved

Abbreviations: AKI: acute kidney injury; CCF: congestive cardiac failure; CKD: chronic kidney disease; d: day(s); ERCP: endoscopic retrograde cholangiopancreatography; LMP: letrozole + micronised progesterone; PE: pulmonary embolism; SAE: serious adverse event; SAE: serious adverse reaction; TIA: transient ischemic attack; TMP: tamoxifen + micronised progesterone; T2RF: type 2 respiratory failure; VQ scan: ventilation perfusion scan.

Supplementary Table 8: Adverse Events Leading to Treatment Discontinuation

AE type	Age at enrolment	Treatment	Days of treatment completed	Severity	Causality*	Summary	Outcome
SAR	76	LMP	12	Mild	Possible	TIA. Pre-existing vascular risk factors	Resolved
AR	72	LMP	8	Mild	Possible	Arthralgia, fatigue, limb oedema, somnolence, headache, breast pain and eye pain	Resolved
AR	65	LMP	9	Mild	Possible	Anorexia, anxiety, gastroesophageal reflux disease, insomnia. Pre-existing depression.	Resolved
AE	90	LMP	1	Mild	Unrelated	Dizziness (onset at 1 hour post first dose of treatment).	Resolved
SAR	86	LMP	3	Severe	Possible	TIA	Resolved
AR	74	LMP	4	Moderate	Probable	Lethargy, ataxia, dizziness	Resolved
AR	76	TMP	5	Mild	Possible	Constipation, limb oedema, fatigue, depression, irritability, paraesthesia, pain in extremity	Resolved
SAE	62	TMP	7	Severe	Unrelated	AKI, diarrhea, nausea, vaginal inflammation, blurred vision, acute infection, haematuria, maculo-papular rash	Resolved

Note: *: investigator assessed relatedness of adverse event to study treatment.

Abbreviations: AE: adverse event, AKI: acute kidney injury; LMP: letrozole + micronised progesterone; SAE: serious adverse event; SAR: serious adverse reaction; TIA: transient ischemic attack; TMP: tamoxifen + micronised progesterone.

Supplementary Figure 1: Correlation Between Ki67 Scored by Each Pathologist

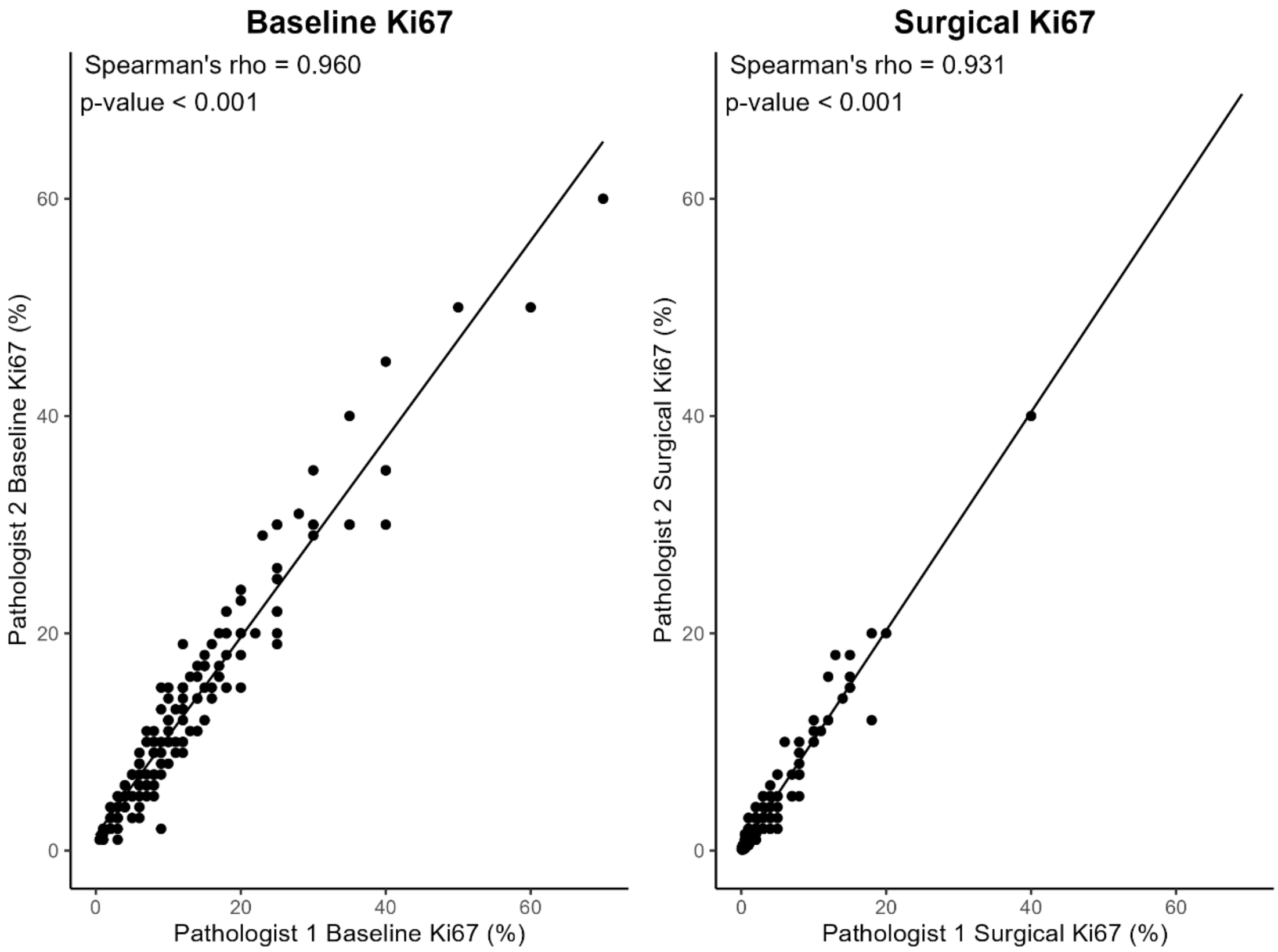

Note: correlation between the Ki67 results of pathologist 1 and 2 were determined using Spearman's rank correlation coefficient (ρ).

Supplementary Figure 2: Change in Geometric Mean

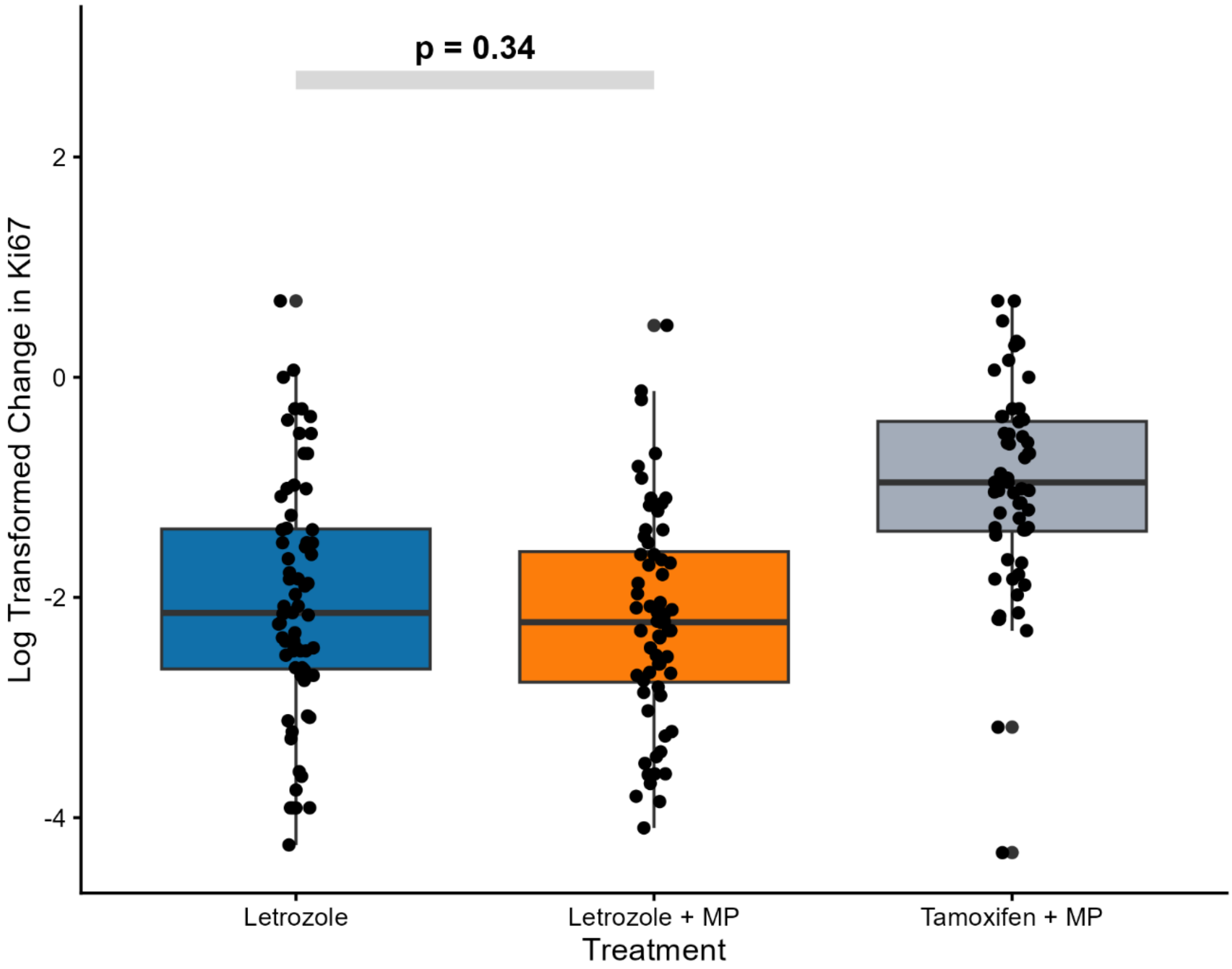

Abbreviations: MP, micronised progesterone.

Supplementary Figure 3: Waterfall Plots of Ki67 Suppression

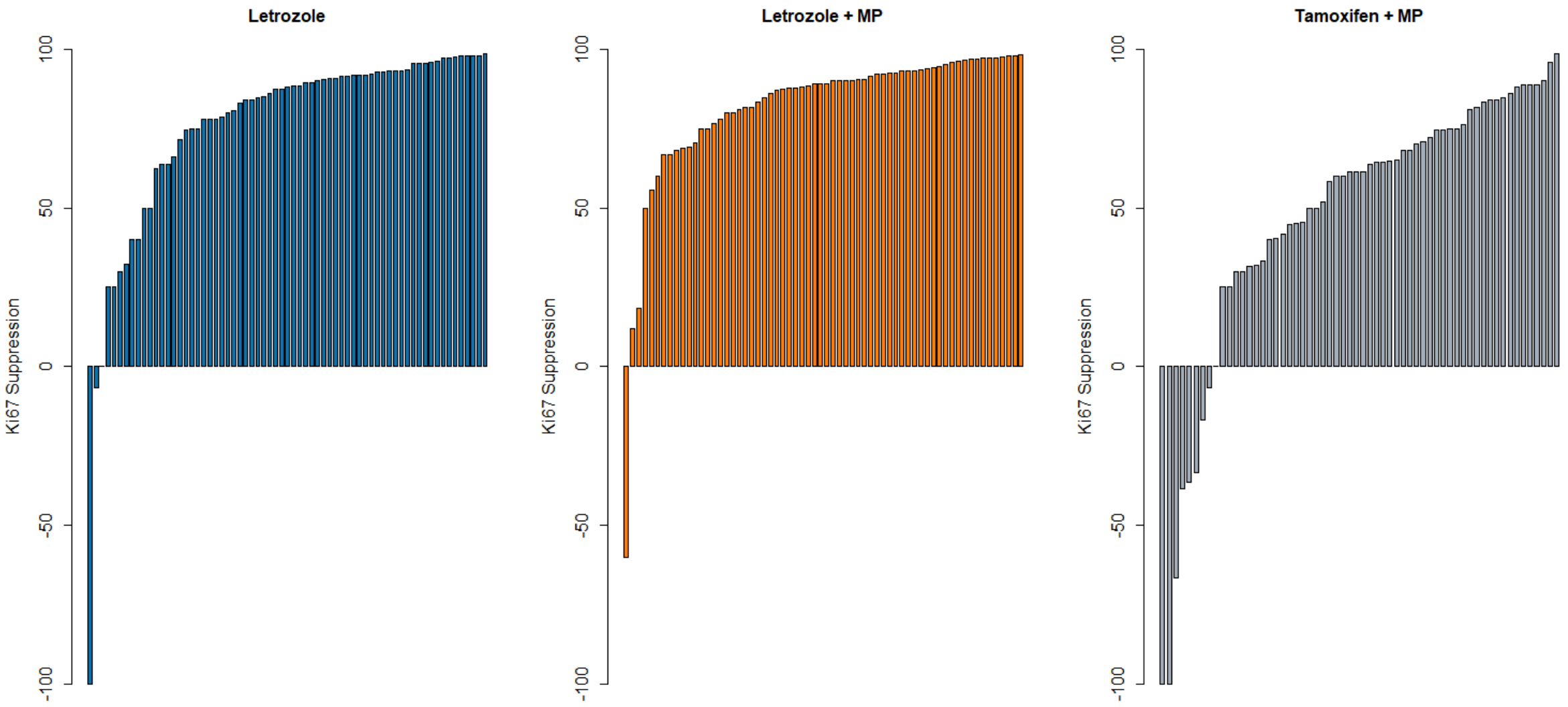

Abbreviations: MP, micronised progesterone.

Supplementary Figure 4: Subgroup Analysis of Ki67 Suppression by Baseline PR Status

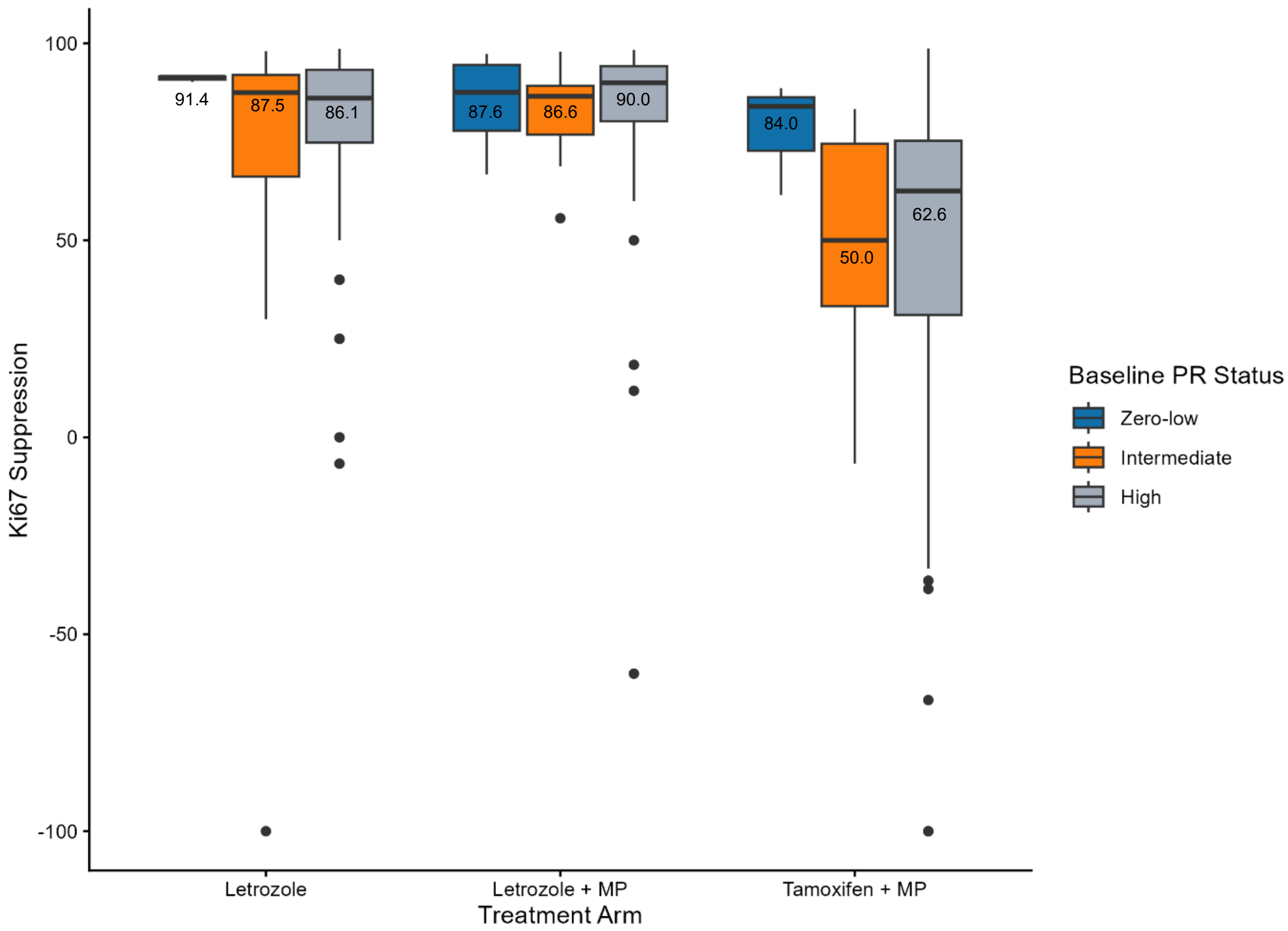

Abbreviations: MP, micronised progesterone; PR, progesterone receptor. Baseline PR Allred scores were grouped into three groups as follows: zero-low if Allred score

was 0-3, intermediate if Allred score was 4-6, and high if Allred score was 8. 137 (72%) of participants were PR high: 47 (25%) in letrozole alone, 46 (24%) in letrozole plus MP, and 44 (23%) in the tamoxifen plus MP groups respectively. 44 (23%) were PR intermediate: 17 (9%), 14 (7%), 13 (7%) in the three groups respectively. 10 (5%) were PR zero-low: 3 (2%), 4 (2%), 3 (2%) in the three groups respectively.

Supplementary Figure 5: Subgroup Analysis of Ki67 Suppression by Baseline Grade

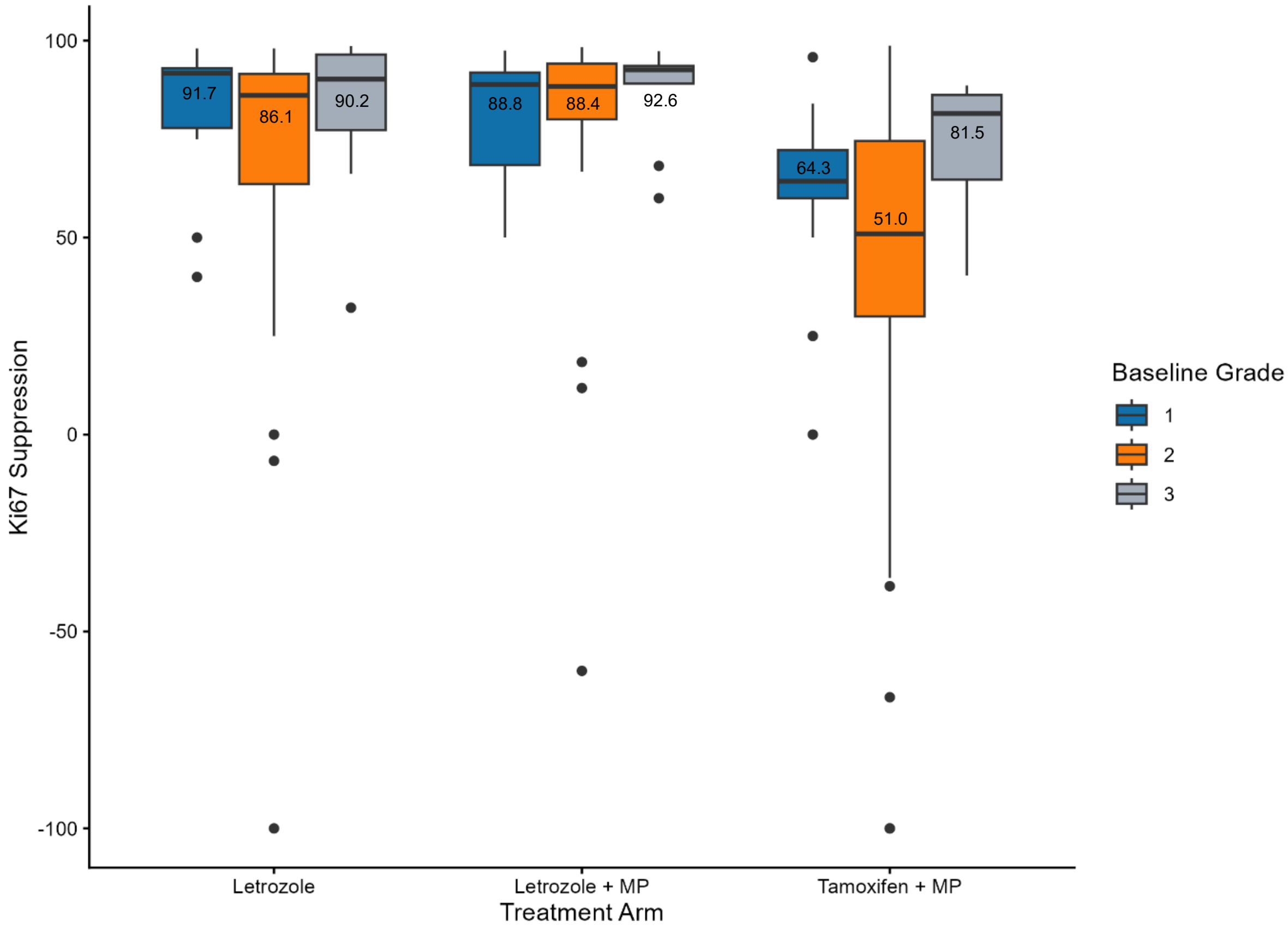

Abbreviations: MP, micronised progesterone. Baseline grade was split into 3 groups based on the centrally scored grade of the cancer. 38 participants (20%) were grade 1: 13 (7%), 12 (6%), 13 (7%) in the letrozole alone, letrozole plus MP and tamoxifen plus MP arms, respectively. 120 participants (63%) were grade 2: 39 (20%), 43 (23%), and 38 (20%) participants in the three arms, respectively. 33 participants (17%) were grade 3: 15 (8%), 9 (5%), and 9 (5%), in the three arms, respectively.

Supplementary Figure 6: Subgroup Analysis of Ki67 Suppression by Baseline Ki67 Status

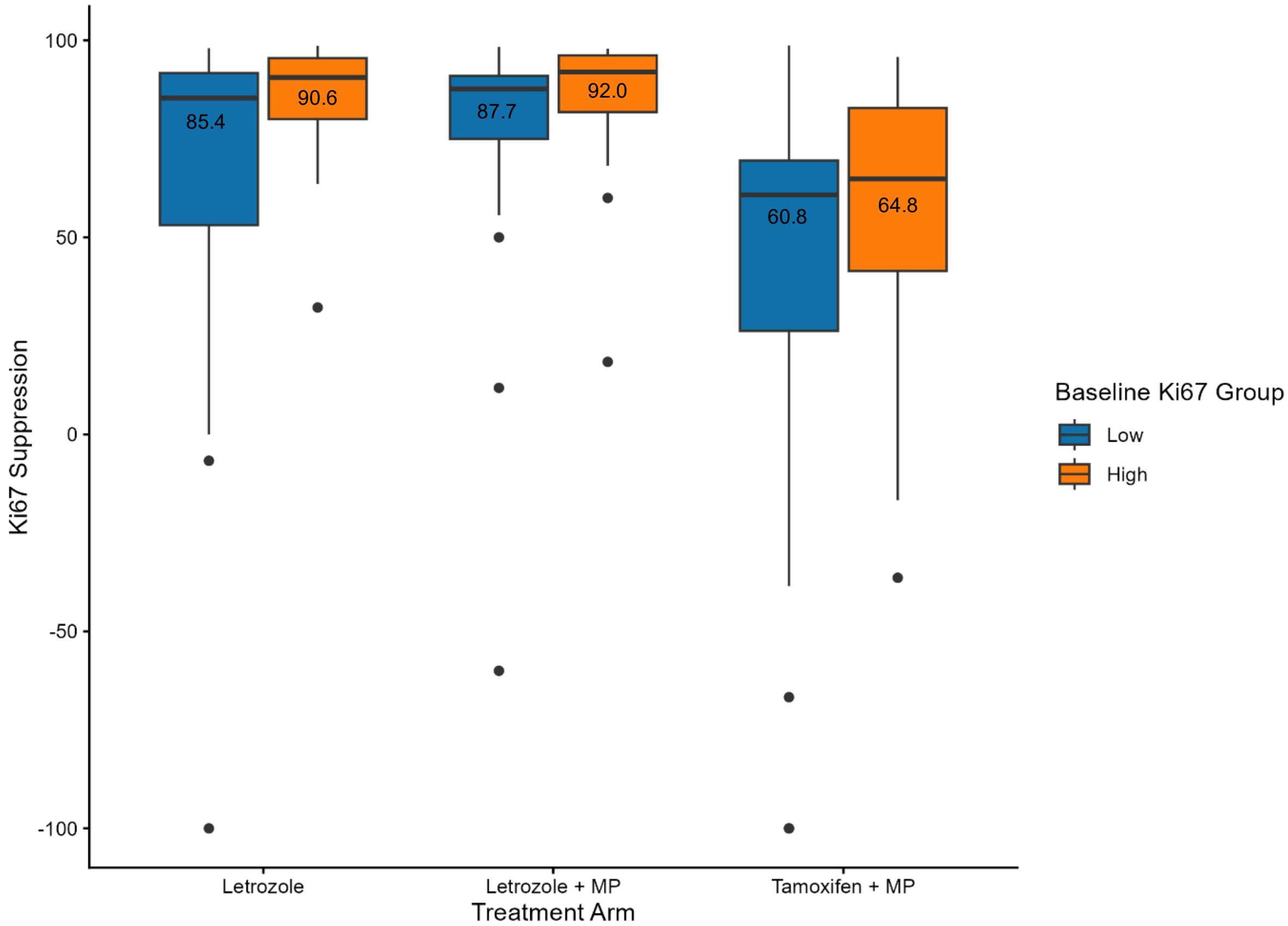

Abbreviations: MP, micronised progesterone. Baseline Ki67 was split into two groups using a cutoff of ≥10% as high and <10% as low. 96 participants (50%) had high Ki67 expression: 29 (15%), 33 (17%), and 34 (18%) participants in the letrozole alone, letrozole plus MP and tamoxifen plus MP arms, respectively. 95 participants (50%) had low Ki67 expression: 38 (20%), 31 (16%), 26 (14%) in the three treatment groups, respectively.

Supplementary Figure 7: Subgroup Analysis of Ki67 Suppression by Histology

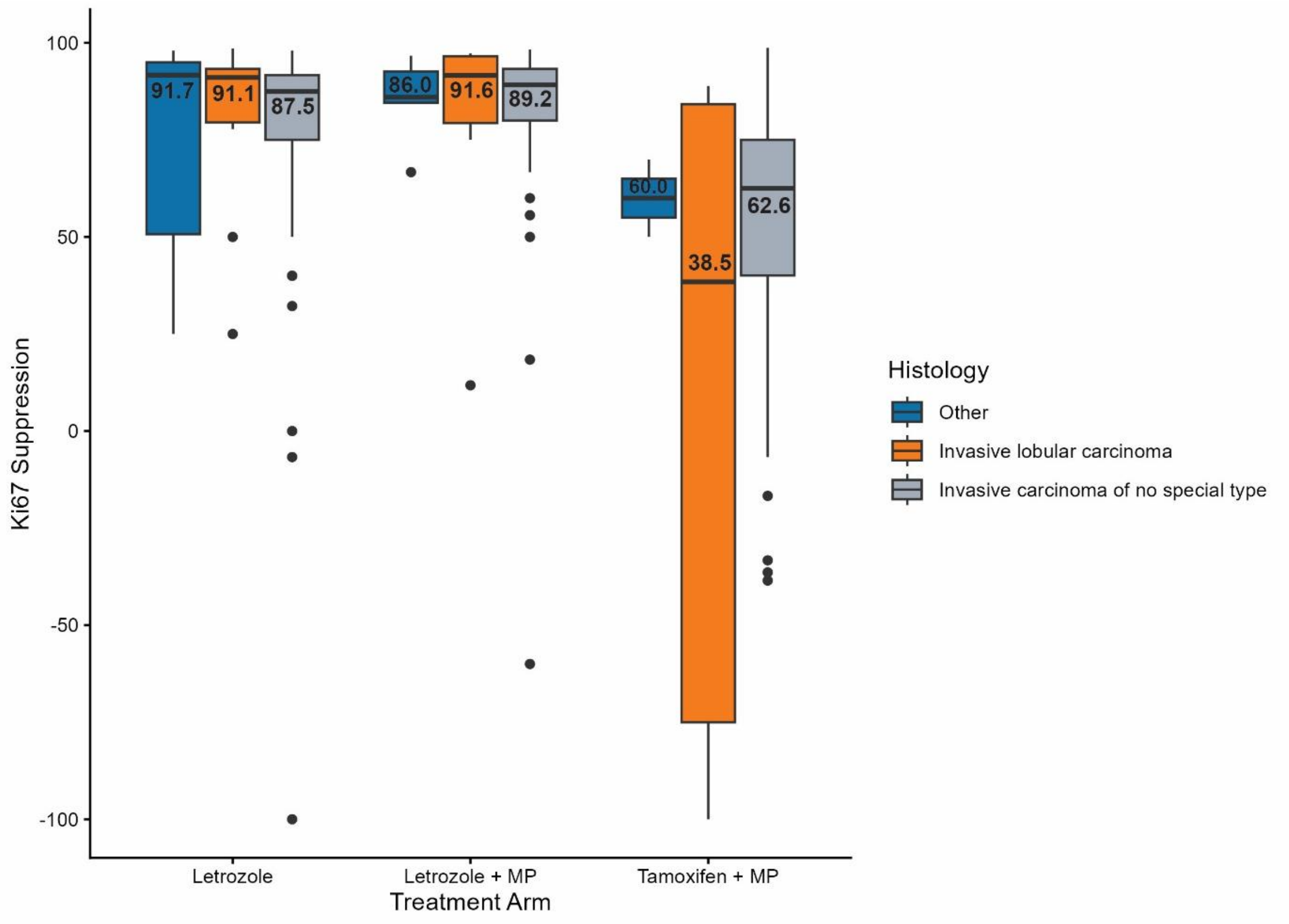

Abbreviations: MP, micronised progesterone. 149 cases (78%) were invasive breast carcinoma of no special type: 50 (26%) participants in the letrozole group, 49 (26%) participants with letrozole plus MP and 50 (26%) with tamoxifen plus MP. 28 cases (15%) were invasive lobular carcinoma: 10 (5%), 10 (5%), 8 (4%) cases in each of the three treatment groups respectively, and 14 (7%) were other subtypes: 7 (4%), 5 (3%), 2 (1%) in the three groups respectively.

Supplementary Figure 8: Final Multiple Linear Regression Model

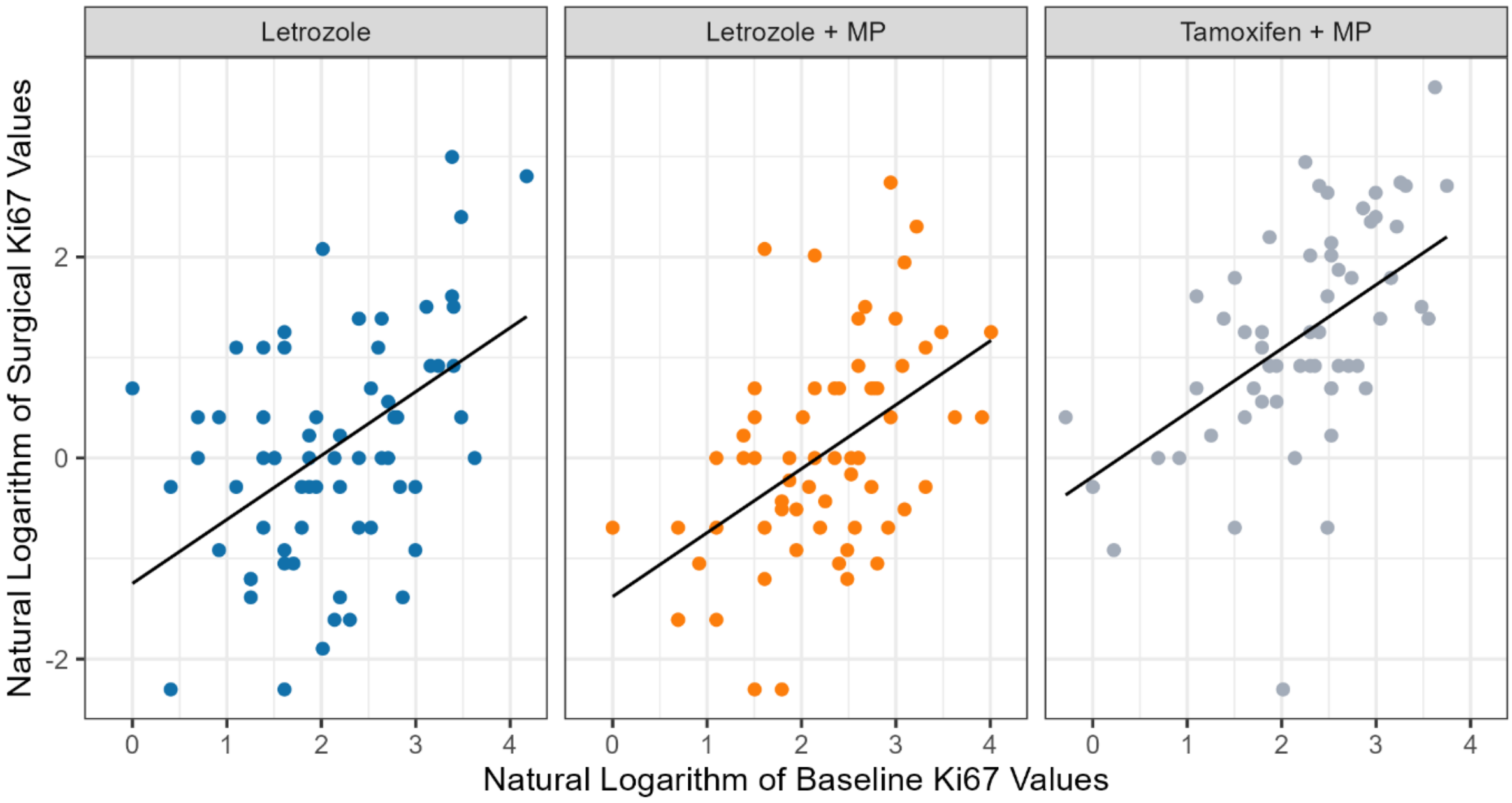

Abbreviations: MP, micronised progesterone.

Supplementary Figure 9: ER and PR Allred Score Suppression by Treatment Arm

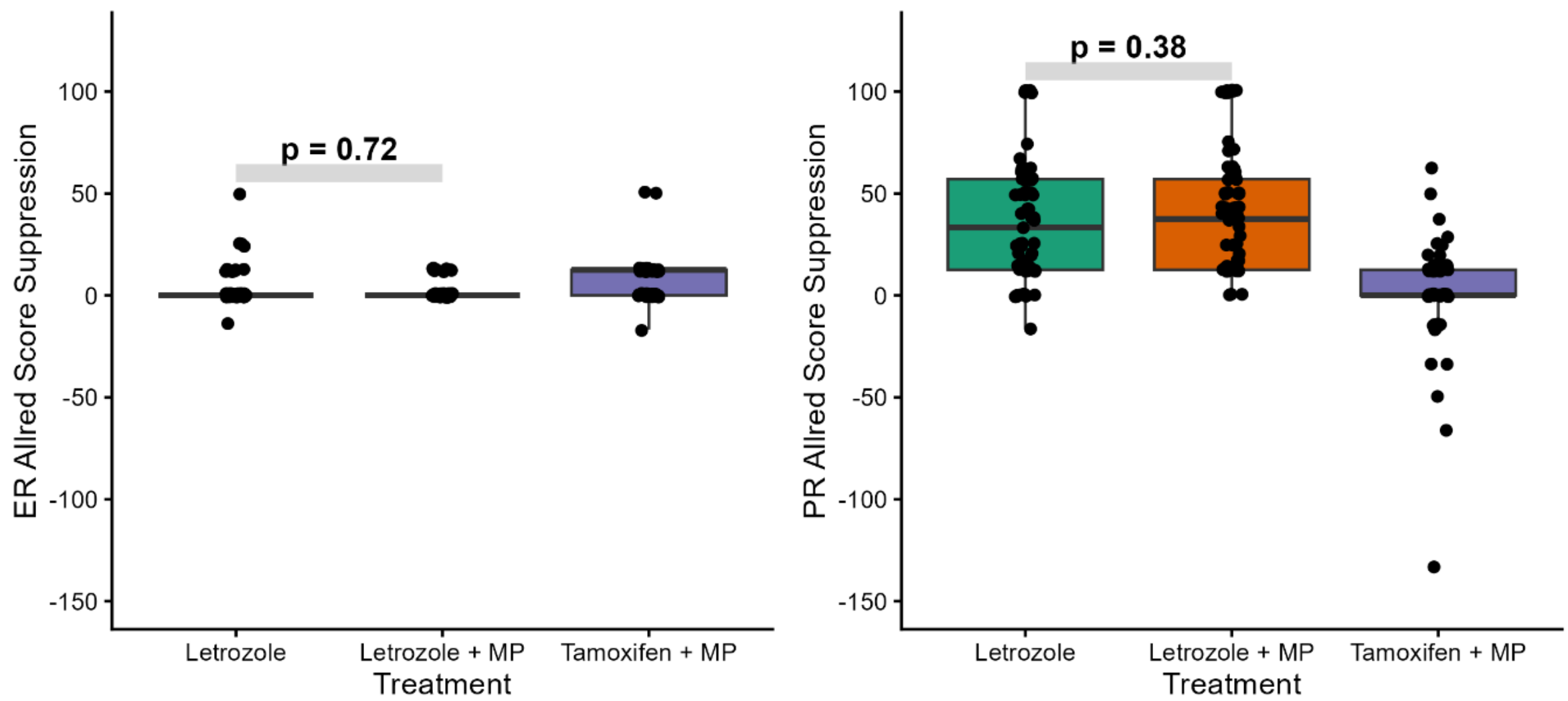

Abbreviations: ER, oestrogen receptor, PR, progesterone receptor, MP, micronised progesterone. The ER and PR Allred score suppressions were calculated as the difference between the post- and pre-treatment Allred scores, divided by the baseline Allred score.

Supplementary Figure 10: Changes in ER and PR Immunohistochemistry

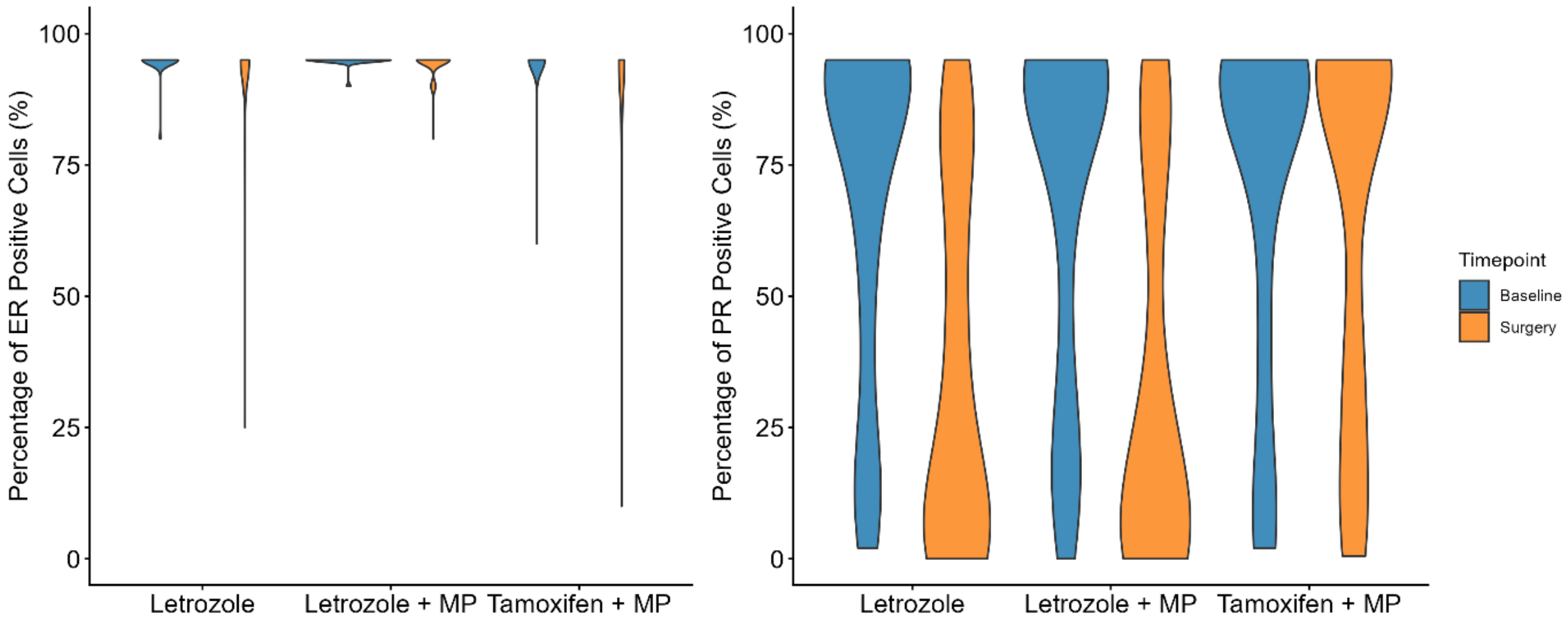

Abbreviations: MP, micronised progesterone.

Supplementary Figure 11: Changes in ER and PR Positivity

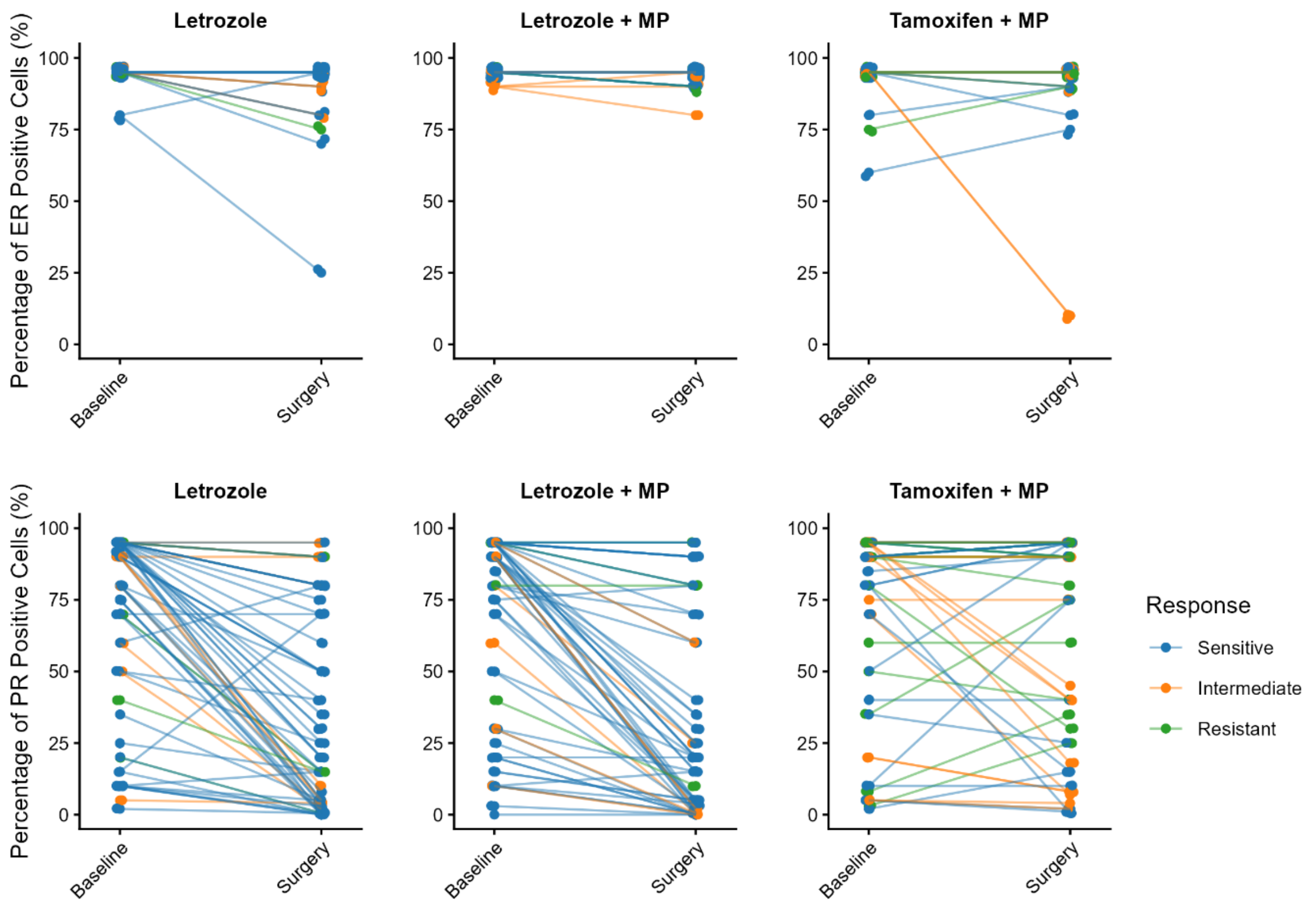

Notes: MP, micronised progesterone. Endocrine therapy response was grouped by the post-treatment Ki67 values as follows: sensitive (≤2.7%), intermediate (>2.7% and <7.4%), resistant (≥7.4%).

Supplementary Figure 12: Adverse Events with Incidence of 1% or Greater in Letrozole Containing Arms

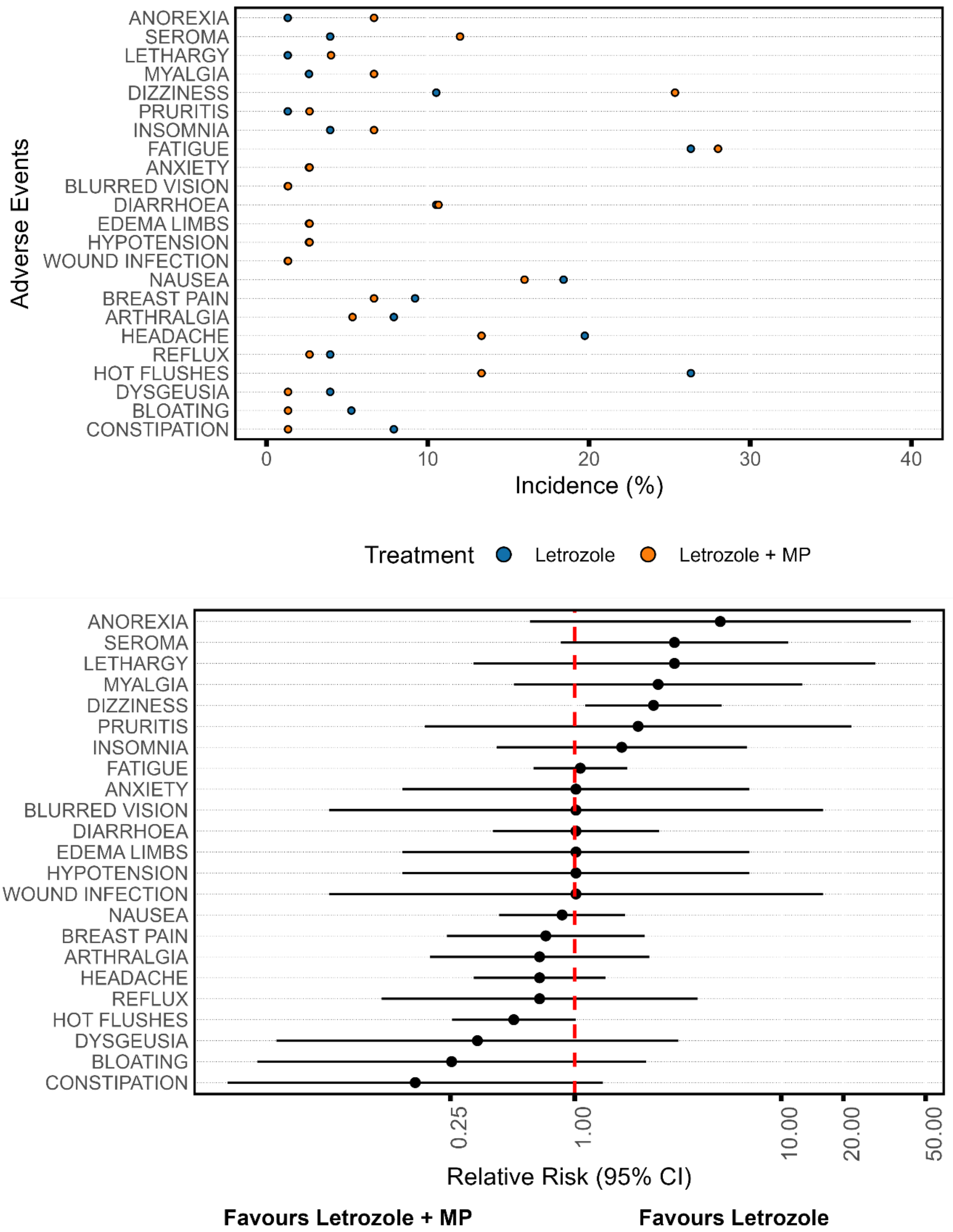

Note: MP, micronised progesterone.

Supplementary Figure 13: Adverse Events with Incidence of 1% or Greater in Micronised Progesterone Containing Arms

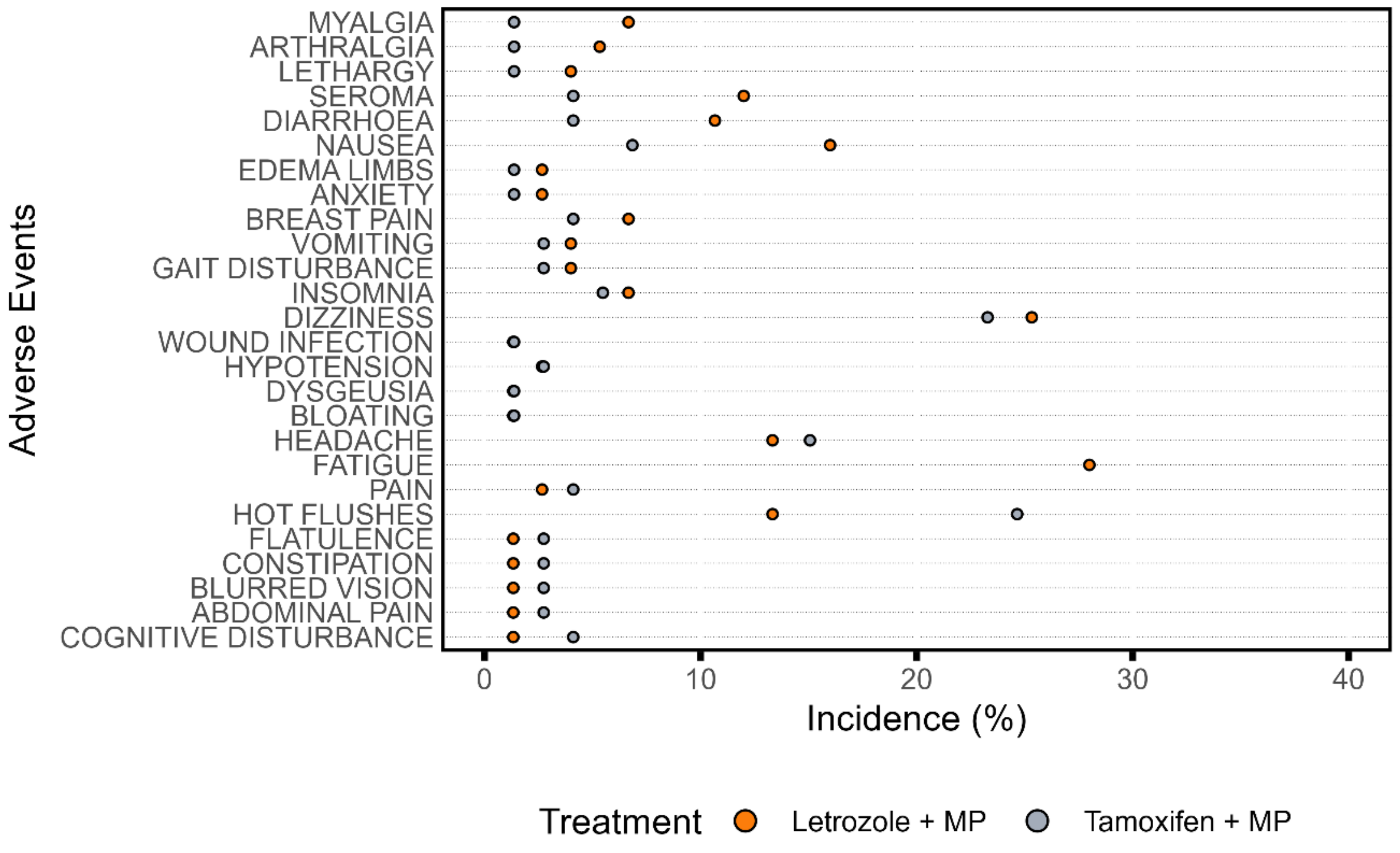

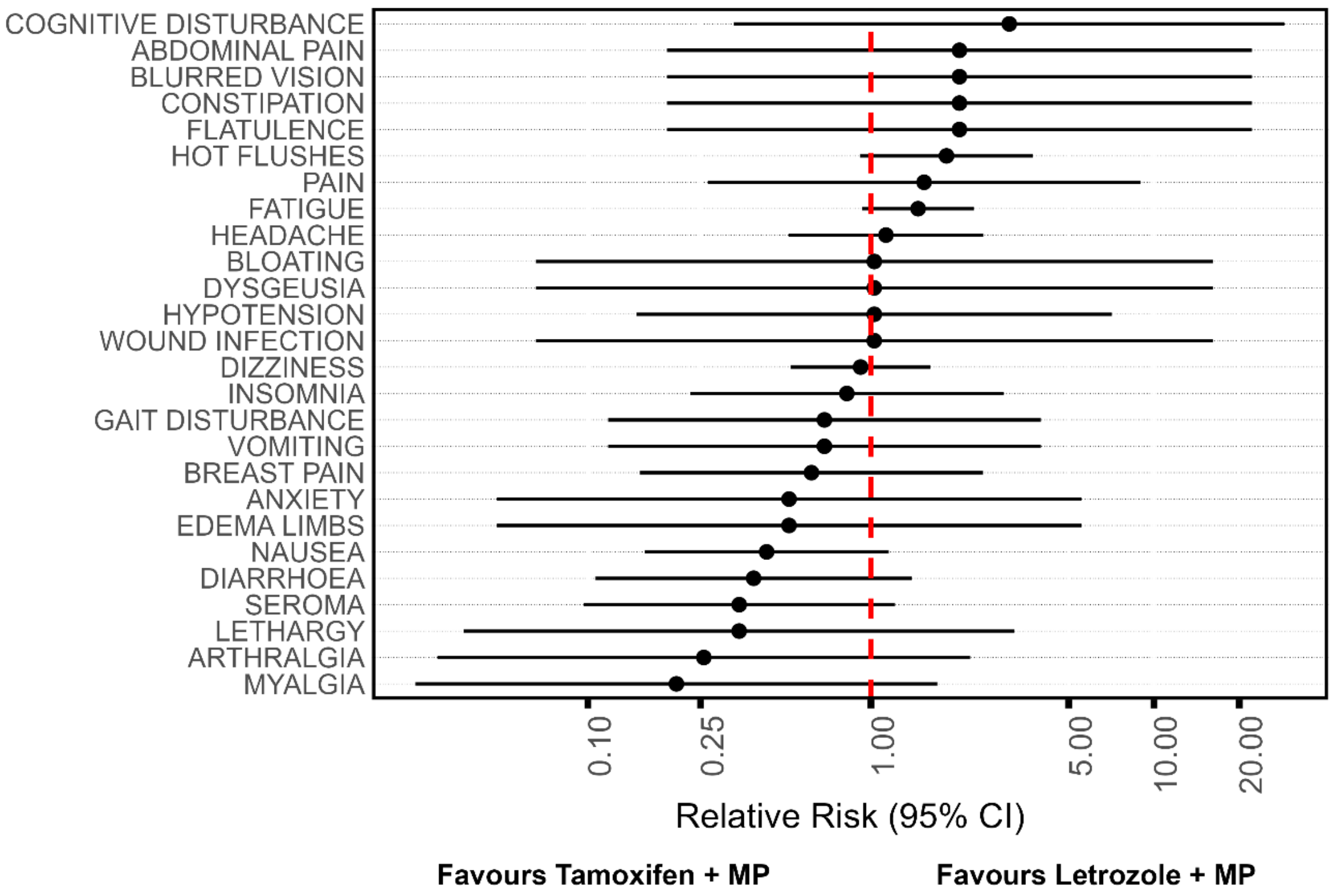

Note: MP, micronised progesterone.

A window of opportunity study of endocrine therapy with and without prometrium in postmenopausal women with early stage hormone receptor-positive breast cancer.
<u>The WinPro Study</u>

Protocol Version 6.0, 06 June 2024

Coordinating Principal Investigator:
Prof Elgene Lim

TABLE OF CONTENT

1. Table of abbreviations
2. Synopsis
3. Study Schema
4. Background and Rationale
5. Aim and Endpoints
6. Design
7. Subject population
8. Intervention Plan
9. Assessment Plan
10. Outcomes and Adverse Events
11. Data and Specimen Collection
12. Statistical Considerations
13. Administrative Aspects
14. References

1. TABLE OF ABBREVIATIONS

ER Estrogen Receptor
HRT Hormone Replacement Therapy
MPA Medroxyprogesterone Acetate
PICF Patient Information and Consent Form
PR Progesterone receptor

2. SYNOPSIS

2.1 Title

A window of opportunity study of endocrine therapy with or without prometrium in postmenopausal women with early stage hormone receptor-positive breast cancer.

2.2 Aim

To assess, in postmenopausal women with early-stage ER+, PR+, HER2-negative breast cancers, the effects of endocrine therapy with or without prometrium on paired ***pre and post-treated breast cancer tissue*** for Cell Proliferation, as measured by tumoral Ki67 expression.

2.3 Hypothesis

We hypothesize that in women with early stage ER+, PR+, HER2-negative breast cancers, intervention with endocrine therapy plus prometrium for 14 days in the window between diagnosis and definite surgery, will result in a significant reduction in cell proliferation (primary end point) when compared to endocrine therapy alone.

2.4 Objectives

2.4.1 Primary Endpoint

The geometric mean suppression of the centrally-assessed proliferation marker Ki67, after two weeks of intervention, compared with baseline. This will be obtained by comparing the mean difference in Ki67 staining between pre and post-treated samples in each intervention arm.

2.4.2 Secondary Endpoints

Safety and tolerability of combination therapy as defined by National Cancer Institute Common Terminology Criteria for Adverse Events (NCI-CTCAE v 4.0).

2.4.3 Translational Endpoints

1. Define a gene set as a predictive biomarker for a reduction in Ki67.
2. Evaluate changes in the apoptotic markers Bcl-2 and Caspase 3 in the breast tumors following intervention.
3. Evaluate changes in ER, PR, AR, FoxA1, Cyclin D1 protein and mRNA expression in the breast tumors following intervention.
4. To determine changes in circulating steroidogenic hormones after two weeks of treatment
5. To determine the difference in circulating hormones using different methods of blood collection
6. To evaluate changes in the tumour microenvironment using spatial transcriptomics.

2.5 Study design

Multi-site, randomised, open-label, three-arm, study in 200 postmenopausal women with early-stage breast cancer between breast cancer diagnosis and surgery.

2.5.1 Intervention and Study Procedures

Eligible postmenopausal women with early-stage ER+, PR+, HER2-negative breast cancer will be randomised 1:1:1 to receive two weeks of intervention with
1) letrozole 2.5mg PO daily,
2) letrozole 2.5mg + prometrium 300mg PO daily, and
3) tamoxifen 20mg + prometrium 300mg PO daily,
between diagnosis of breast cancer and definite surgery. The intervention treatment should be administered in the 14 days (+/- 3 days) directly before the planned surgery date, e.g. day 14 will be the day before surgery, day 15 will be the day of surgery. Following surgery, patients will receive standard adjuvant therapy as recommended by their treating team.

Postmenopausal status is defined by any one of the following criteria;
a) Women ≥ 50 years with amenorrhea >12 months at the time of diagnosis and an intact uterus
b) Women of any age with prior bilateral oophorectomy, and/or
c) Women < 50 years with FSH and estradiol levels within the postmenopausal range (as per local practice)

Prometrium® (Besins Healthcare) is a bioidentical micro-ionised progesterone that was developed from yams, is chemically identical to progesterone of human ovarian origin, and is FDA-approved for use with estrogen drugs for the prevention of endometriosis at a dose of 200-300 mg daily (1). It is also used on its own as a treatment for menopausal symptoms. It is made as a capsule that contains microionized progesterone for oral administration, and is metabolized in the liver.

2.6 Sample size

Minimum of 200 evaluable women.

2.8 Study Duration

36 months

3. STUDY SCHEMA

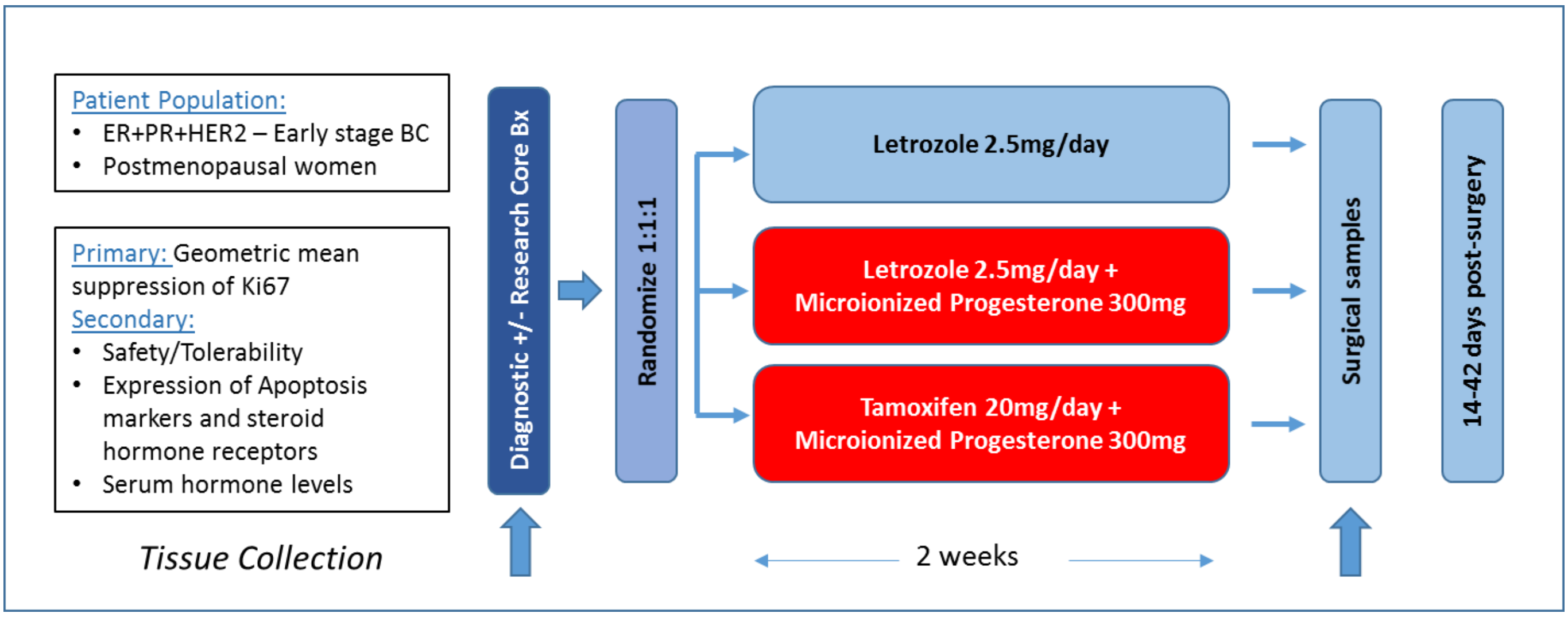

4. BACKGROUND AND RATIONALE

Breast Cancer is a heterogeneous disease affecting ≈15,000 Australian women each year (3). ≈75% of breast cancers expresses the nuclear transcription factor estrogen receptor α (ER) (4). The majority of ER+ breast cancers also express other steroid hormone receptors, including the Progesterone Receptor (PR), an ER-induced gene and a well-established biomarker of ER functionality. ER+ breast cancers are associated with better outcomes and increased sensitivity to endocrine therapy when they co-express PR (5,6).

The role of progestins in normal breast tissue and breast cancer is controversial. Synthetic progestins widely used in hormone therapies to treat menopausal symptoms, are linked to an increased risk in breast cancer (7). In contrast, native progesterone resulted in no change, or a slight decrease in breast cancer incidence (8-12). Clinical trials of progestins such as megestrol acetate (megace) and Medroxyprogesterone Acetate (MPA) have demonstrated consistent benefit in women with advanced ER+ breast cancers (13-17). Unlike ER, the functional role of PR in breast cancer has been poorly understood up to now. Given that nuclear receptors have similar consensus DNA binding motifs and commonly use the same co-factors for activity (18), it is not surprising that substantial cross-talk occurs between nuclear receptors in cancer. Activation or inhibition of parallel hormonal pathways, may therefore, impact on the steroid hormone receptors responsible for driving tumour growth.

We have recently described substantial interplay between PR and ER in human breast cancers (19). We demonstrated that PR activity can reprogram where ER binds to DNA, directly modulating ER function, resulting in changes in gene expression profiles that cumulated in cell cycle arrest. The combination of PR agonists with tamoxifen showed greater inhibitory effects in breast cancer in vitro and in vivo than either agent alone. The clinical implications of these seminal findings are that progesterone may be used to enhance the efficacy of current endocrine therapies.

Here, we propose to translate our preclinical findings by conducting an open-label, pilot, window of opportunity study between diagnosis and definite surgery, to assess the effect of tamoxifen in combination with microionized progesterone (prometrium) in 30 patients with newly diagnosed ER+PR+ breast cancer. The major aim of our study is to assess the effect of such intervention on pre and post intervention tissue samples, on markers of proliferation. This study will provide the critical clinical evidence of efficacy in adding progesterone to endocrine therapies in patients with early stage breast cancer, allowing us to move forward in a preoperative systemic treatment clinical trial in a larger patient cohort.

4.1 Hypothesis

We hypothesize that in postmenopausal women with early stage ER+, PR+, HER2-negative breast cancers, intervention with prometrium plus tamoxifen in the window between diagnosis and definite surgery, will result in a significant reduction in proliferation (primary end point) when compared to anti-estrogen interventions alone.

4.2 Significance

The outcomes of this study will have significant clinical relevance for patients with ER+ and PR+ breast cancers, which represent the most common subtype of breast cancer. It builds on seminal preclinical work by co-investigators W Tilley and J Carroll, demonstrating the cross-talk between ER and PR in estradiol-responsive breast cancers, and the additive anti-cancer effect with the addition of natural progesterone to endocrine therapy. We hypothesize that the combination therapies will be more effective than current standard of care at arresting tumour growth and stimulating a gene expression profile that drives a good clinical outcome. We aim to provide the necessary early clinical evidence that an interesting laboratory finding warrants testing in a broader clinical setting. This window of opportunity study will accrue quickly and its short two-week design, established in the context of endocrine therapies in ER+ and PR+ breast cancers, will allow for the evaluation of the efficacy of adding progesterone to endocrine therapies. Micro-ionized progesterone can be considered a repurposed drug as it is used primarily in the setting of treating postmenopausal symptoms. Should this study be positive, it would add a relatively inexpensive treatment option to women with hormone receptor-positive breast cancer.

The collection of paired pre and post-treated samples represent an invaluable resource for researchers to further understand how hormonal manipulation affects breast cancer, the identification of new targets that may be manipulated for therapeutic benefit in these breast cancers, presenting opportunities for new targeted preclinical drug development.

This study has the potential to expand the proportion of breast cancer patients in whom endocrine therapy can be effective. If our end points are met, it provides the critical rationale to move this novel therapeutic concept into a larger preoperative systemic trial of a longer duration, or a study in metastatic breast cancer. Our findings will inform the design and patient selection of clinical trials exploiting PR as a therapeutic modality in ER+ and PR+ breast cancers.

5. AIM AND ENDPOINTS

5.1 Aim

To assess, in postmenopausal women with early-stage ER+, PR+, HER2-negative

breast cancers, the effects of endocrine therapy with or without prometrium on paired ***pre and post-treated breast cancer tissue*** for Cell Proliferation, as measured by tumoral Ki67 expression.

5.2 Endpoints

5.2.1 Primary Endpoint

The geometric mean suppression of the centrally assessed proliferation marker Ki67, after two weeks of intervention, compared with baseline. This will be obtained by comparing the mean difference in Ki67 staining between pre and post-treated samples in each intervention arm.

5.2.2 Secondary Endpoints

Safety and tolerability of combination therapy as defined by National Cancer Institute Common Terminology Criteria for Adverse Events (NCI-CTCAE v 4.0).

5.2.3 Translational Endpoints

1. Define a gene set as a predictive biomarker for a reduction in Ki67.
2. Evaluate changes in the apoptotic markers Bcl-2 and Caspase 3 in the breast tumors following intervention.
3. To determine the difference in circulating hormones using different methods of blood collection
4. 4. To evaluate changes in gene expression that occur in response to endocrine therapy in tumoral cells and in the tumour microenvironment using spatial transcriptomics, and to correlate these changes with Ki67 response to endocrine therapy.

<u>6. STUDY DESIGN</u>

Multi-site, randomised, open-label, three-arm, study in 200 postmenopausal women with early-stage breast cancer between breast cancer diagnosis and surgery.
Eligible postmenopausal women with early-stage ER+, PR+, HER2-negative breast cancer will be computer randomised 1:1:1 to receive two weeks of intervention with:
1) letrozole 2.5mg PO daily,
2) letrozole 2.5mg + prometrium 300mg PO daily, and
3) tamoxifen 20mg + prometrium 300mg PO daily,
between diagnosis of breast cancer and definite surgery. The intervention treatment should be administered in the 14 days (+/– 3 days) directly before the planned surgery date, e.g. day 14 will be the day before surgery, day 15 will be the day of surgery.
Following surgery, patients will receive standard adjuvant therapy as recommended by their treating team.

<u>7. SUBJECT POPULATION</u>

7.1 Inclusion Criteria

a) Postmenopausal status as defined by one of the following criteria
   I. Women > 55 years with amenorrhea >12 months at the time of diagnosis and an intact uterus
   II. Women of any age with prior bilateral oophorectomy, and/or
   III. Women < 55 years with FSH and estradiol levels within the postmenopausal range (as per local practice)

b) Histologically confirmed ER+ ***and*** PR+ breast cancers (defined as ≥10% positive staining cells determined by local pathological assessment).
c) HER2-negative, defined as:
    I. Immunohistochemistry (IHC) 0 or 1+; or
    II. IHC 2+ and pending ISH; if subsequent ISH (in situ hybridization) is positive, patient will be excluded from efficacy analysis; or
    III. IHC 3+ and ISH negative
d) Tumour size ≥5 mm as measured by any one of; clinical assessment, ultrasound ,mammogram or MRI.
e) Ability to understand all patient information and informed-consent documents, written informed consent to participate in the trial, and to avail tissue and blood samples for research.
f) Aged 18 years or older.

7.2 Exclusion Criteria

a) Women currently on hormone therapies, including HRT and OCP.
b) Locally advanced and inoperable, or inflammatory breast cancer.
c) Clinical evidence of metastatic disease.
d) Patients treated with other preoperative systemic therapies.
e) Nut allergy (prometrium contains peanut oil).
f) Prior history of uterine cancer, deep vein thrombosis, pulmonary embolism or clotting disorder.
g) Women who are pregnant or breast feeding.

7.3 Consent

Informed consent will be obtained from the participant during their appointment with the breast surgeon co-investigator, following the diagnosis of their breast cancer. The participants will be given the PICF to take home, and inform their surgeon of their intention to participate or not. Potential participants will be reminded that declining to enroll into the study will not have any negative impact on their subsequent care and that participation is voluntary. Participants will then be given the opportunity to ask questions of the investigator prior to giving informed consent and enrolling into the study.

Once a participant gives informed consent and signs the PICF, the investigators will document this in the participant's medical record, including the participants study enrolment number.

7.4 Randomisation

Upon meeting the inclusion and exclusion criteria, and signing of the PICF, the patients will be registered in the study electronic data capture system (EDC). The participant will receive a study enrolment number. The patient will be randomised to the treatment group using the EDC randomisation module. A printout of the treatment group will be retained in the patients' medical record.

8. STUDY PLAN

Following a new diagnosis of breast cancer, the investigators will consent appropriate patients who meet the inclusion and exclusion criteria. Confirmation of signed consent (without identifying details), inclusion and exclusion criteria checklist,

and proposed date of surgery will be confirmed electronically and forwarded to the Kinghorn Cancer Centre, St Vincent's Hospital Sydney coordinating team The medical oncologist will discuss the study with the patient, prescribe the drugs, and give directions as to how to take the study drugs. Upon approval by the primary investigator, medications will be dispensed adhering to the legal framework for drug dispensing. The patients will have their primary surgery and subsequent adjuvant systemic therapy as standard of care. A follow up and end of study visit will be made at 2 to 6 weeks post-surgery with the site investigator to assess if they have any reportable side effects. Study visits and intervention should be carried out within the window described in section 9.1 Patients who cannot meet this plan will be considered non-evaluable.

The tissue used for the primary end point of this study is the diagnostic biopsy (pre-intervention), and the surgical sample (post-intervention). This will be in the form of formalin fixed paraffin embedded (FFPE) tissue. 20 unstained slides will be requested from each of these tissue blocks for central analysis by immunohistochemistry. In addition, fresh frozen material will also be obtained from the surgical tissue at sites with tissue banking facilities. Patients enrolled at these sites will also be approached for an optional research biopsy to be taken prior to the initiation of therapy. This will involve an additional biopsy to the diagnostic biopsy, which will be fresh frozen for RNA analysis.

8.1 End of Study
Data to collect at the end of study visit include: adverse events, surgery type/details, histopathology, surgical complications and subsequent cancer therapies.

9. ASSESSMENT PLAN

9.1 Schedule of trial visits, interventions and measurements

	**Screening**	**Baseline**	**Contact**	**Surgery**	**End of study**
	No limit	D -7 to 0	D 5-10	D 12-18	D 14-42 (post-surgery)
**Patient consent**	X				
**Tumour tissue collection**	X			X	
**Blood collection**[a]		X		X	
**Clinical review**		X[c]			X[c]
**Adverse Events/Compliance**[b]			X	X	X[d]

[a] Finger prick and venous blood collection.
[b] Telephone or clinic contact to confirm compliance and monitor Adverse Events.
[c] As per local standard.
[d] Note that while the end of study visit may occur between days 14-42 post-surgery, as per Section 10.2 on page 12, adverse events are to be collected collected and recorded only up to 14 days post-surgery.

9.2 Assessment of biomarkers by Immunohistochemistry

20 unstained slides will be requested from the pathologist of the paired initial diagnostic biopsy and surgical sample for analysis of the primary and translational endpoints. Ki67 in the FFPE tissue will be analysed centrally in a National Association of Testing Authorities Australia (NATA) accredited diagnostic laboratory (Sydpath) by two experienced breast cancer pathologist (*Co-investigators Sandra O'Toole and Andrew Parker)*, from core biopsies collected at baseline (pre-

intervention) and at two weeks (time of surgery). It will be measured according to the international working group guidelines using the MIB1 anti-human Ki67 monoclonal antibody (20), with numeration of at least three randomly selected high power fields (21).

In addition, other proteins may be assessed, including apoptotic markers Bcl-2 (clone 124, Dako) and cleaved-caspase 3 (clone D175, Cell signalling), steroid hormone receptors ER (clone SP1), PR (clone IE2), AR (clone 441, Dako), Cyclin D1 (clone SP4, Thermo Fisher) and FoxA1 (Abcam ab23738), all assessed via a H score combining percentage of tumour cell nuclei and intensity of expression, will also be centrally analysed in the pre and post intervention samples.

9.3 RNA analysis by quantitative PCR

RNA will be extracted from the fresh frozen material in CIB Tilley's laboratory with Qiagen AllPrep RNA extraction kits. The RNA will then be analysed by qPCR for ER, PR, AR and other important nuclear steroid receptor cofactors such as FoxA1. The RNA may then also be used for the generation of labelled cDNA and subsequently used for RNA-sequencing to determine the differences in expression of genes between the paired samples in the two intervention arms of the trial.

9.4 Development of Progesterone gene set as a predictive biomarker for response to prometrium

In the previous study published in Nature, 470 genes were differentially regulated by dual treatment with estrogen and a progestogen compared to estrogen alone in breat cancer cell lines (19). There was a pronounced enrichment of progestogen-induced ER binding events near genes upregulated by progestogen treatment in the presence of estrogen, indicating that the re-programming of ER to new sites on chromatin by PR results in new gene expression profiles. Importantly, the gene signature driven by progesterone-stimulated ER binding was associated with good prognosis in a large cohort of women.

The material generated in this window trial will be used to validate the "good outcome" gene set that results from reprogramming of ER by PR. We will refine the gene signature to identify the core set of genes that contributes to good outcome. The signature will be further refined by integration with ER and PR genome-wide DNA binding profiles. Specifically, we will prioritise genes with a proximal "reprogrammed" binding event (i.e. a shared ER/PR binding site) that is evident only in the presence of estrogen and progesterone. Expression of the refined "good outcome" gene signature (up to 50 genes) will be tested in the pre- and post-intervention tissues from our study using the Nanostring nCounter system, which allows for precise quantification of multiplexed RNA and functions well with low amounts total RNA (≥100ng). We hypothesize that the signature will be elevated post-intervention in patients that respond to prometrium. We will also test whether expression of the signature pre-intervention is a predictive marker of response in our trial, since we expect that progesterone will only be able to elicit protective effects if the tumour is sensitive to PR ligands.

9.5 Serum studies

The trial will collect serum to study changes in the serum hormonal levels and bone remodeling markers. Dr Hubert Vesper, our collaborator at the National Institute of

Health (USA) has developed a high sensitivity isotope dilution liquid chromatography tandem mass spectrometry (ID LC- MS/MS) method to measure total Testosterone (T), dihydro-testosterone (DHT), estrone (E1), estradiol (E2), progesterone (P4), and Dehydroepiandrosterone Sulfate (DHEAS) in serum. This will ensure accurate and robust measurements of T and E2 and their biologically active metabolites, allowing evaluation of known androgen metabolite effects (41). We will collect serum from patients recruited and these serum studies may be done if funding for this component of the project is secured. We will also collect finger prick dried blood using a unique hormone assay developed by Dr David Zava of ZRTlabs to assess the hormone levels and compare these with hormone levels assessed using venous blood.

9.6 Spatial Transcriptomics

This study will evaluate changes in gene expression that occur in response to endocrine therapy in tumoral cells and in the tumour microenvironment using spatial transcriptomics. Xenium In Situ technology (10x Genomics) will be used to conduct the spatial transcriptomic analyses. This technology enables the detection and evaluation of the transcriptome on a cellular resolution, and localises this data in a spatial context. This data provides important insights into the structure and function of cancer cells and the surrounding tumour microenvironment. The data will be analysed using bioinformatic approaches. These changes will also be correlated with Ki67 response to endocrine therapy.

10. OUTCOMES AND ADVERSE EVENTS

10.1 Outcomes

The outcomes of this study will have significant clinical relevance for patients with ER+ and PR+ breast cancers. It builds on seminal preclinical work by *co-investigators W Tilley and J Carroll*, demonstrating the cross-talk between ER and PR in estradiol-responsive breast cancers, and the additive anti-cancer effect with the addition of natural progesterone to endocrine therapy (19). We hypothesize that the combination therapies will be more effective than current standard of care at arresting tumour growth and stimulating a gene expression profile that drives a good clinical outcome. We aim to provide the necessary early clinical evidence that an interesting laboratory finding warrants testing in a broader clinical setting. This window of opportunity study will accrue quickly and its short two-week design, established in the context of endocrine therapies in ER+ and PR+ breast cancers, will allow for the evaluation of the efficacy of adding progesterone to endocrine therapies. Microionized progesterone can be considered a repurposed drug as it is used primarily in the setting of treating postmenopausal symptoms. Should this study be positive, it would add a relatively inexpensive treatment option to women with hormone receptor breast cancer, the largest subtype of breast cancer.

10.2 Adverse Events

Prometrium is safe and well tolerated, and at a dose of 300mg, no differences in adverse effects as compared to placebo were seen (22, 23). While there are no data on the concurrent administration of tamoxifen and prometrium, tamoxifen has been given safely concurrently with other progestins such as MPA and megace (24, 25).

Adverse Events (AE's) will be acted upon and documented at each visit, from the time of treatment commencement. Participants will be withdrawn if they have a change in safety assessments that necessitate clinical intervention.

Serious Adverse Events (SAE's), regardless of whether related or unrelated to the study drugs, must be reported to The Kinghorn Cancer Centre, St Vincent's Hospital within 24hrs of the site investigators knowledge of this event, from the time of treatment commencement.

Adverse Events of Special Interest (AESI's) include bleeding complications, see point 10.2.1. AESI's should be reported to the Kinghorn Cancer Centre, St Vincent's Hospital within 48hrs of learning of the event.

Serious Adverse Events and Adverse Events are reported in line with NHMRC guidance: Safety monitoring and reporting in clinical trials involving therapeutic goods November 2016, and should be actioned upon as per local institutional guidelines and collected/recorded up to 14 days post-surgery.

10.2.1 Venous Thromboembolism Risk and Management

Two weeks of letrozole, tamoxifen or prometrium treatment carries a low risk of venous thromboembolism (VTE). The risk of combined endocrine therapy is unknown.

There is a low theoretical risk of increased risk of VTE with combination of the two drugs, as both tamoxifen and synthetic progestin containing HRT are associated with a small increased risk of DVT, and are associated with a prolonged treatment period. This was not reported in the trials of MPA and megace in combination with tamoxifen. Importantly, MPA is a synthetic progesterone in contrast to prometrium.

<u>Assessing VTE risk</u>

All patients should be assessed for VTE risk. Patients with a personal history of VTE (including deep venous thrombosis and pulmonary embolism) or a clotting disorder should be excluded from the study.

<u>Prescribe appropriate VTE prophylaxis with surgery</u>

All patients should undergo a VTE risk assessment by the surgeon and if appropriate, be prescribed pharmacological and/or mechanical prophylaxis. Routine perioperative VTE prophylaxis includes enoxaparin 40mg daily or heparin 5,000 international units twice daily plus mechanical thromboprophylaxis.

The use of prophylactic anti-coagulation can increase the risk of bleeding complications intra-operatively and post-operatively. The use of prophylactic anti-coagulation will be at the discretion of the local surgeon, in accordance with local site health service guidelines.

11. DATA AND SPECIMEN COLLECTION

A Clinical Research Monitor will coordinate the study and provide data verification according to a monitoring plan.

The biopsy and surgical specimens will be collected at the local sites of the study. 20 unstained slides will then be requested from each paired tissue sample and sent to the central laboratory for staining in batches.

Research data regarding the patient and tumour information will initially be collected and stored at the local institution. Research data will then be periodically transferred to the eCRF at the Kinghorn Cancer Centre. Research data will be stored in a re-identifiable format (to which the identifying key will remain at the participant's local institution only) and retained for a minimum of five years from the date of publication. This research data will be transferred to the Garvan Institute of Medical Research for analysis by Dr Lucy Haggstrom and Prof Alan Coates, with oversight from the CPI.

12. STATISTICAL CONSIDERATIONS

Statistical analysis of the study will be overseen by Prof Alan Coates and Prof Val Gebski. A review of the literature suggests that the IMPACT study remains the best data set with which to compare our pilot experience (20,21). They reported a geometric mean reduction in Ki67 after 2 weeks of preoperative tamoxifen of 59.5%, and anastrazole of 76%.

There are three potential comparisons:
1. Letrozole alone vs letrozole + Prometrium;
2. Letrozole alone vs tamoxifen + Prometrium; and
3. Letrozole + Prometrium vs tamoxifen + Prometrium

This allows estimation of power to detect differences between arm 1 and either arm 2 or arm 3. For the third possible comparison (Arm 2 vs Arm 3) there is no obvious prior evidence. We will therefore treat this as a purely exploratory comparison and test the other 2 at a p-value of 0.025 each. With a total trial recruitment of 200 and allowing 4% dropouts, this would give 80% power to detect an improvement in Ki67 suppression from 76% in the letrozole alone control arm to 92% in either experimental arm.

13. ADMINISTRATIVE ASPECTS

13.1 Study Structure

This is a multi-site, randomised, open-label, three-arm, study in 200 postmenopausal women with early-stage breast cancer between breast cancer diagnosis and surgery.

13.2 Funding

This study is funded by grants from the Cancer Council of NSW (CIA Lim) and the NHMRC Translational Breast Cancer Project grant (CIA Lindeman).

13.3 Ethics and regulatory compliance

Approval for ethical aspects of this research project has been granted by the HREC of St Vincent's Hospital, Sydney. The study will be performed in accordance with the principles laid down by the World Medical Assembly in the Declaration of Helsinki 2004. Site-specific Governance approval will be sought for all participating sites.

Participants will be given a full and clear explanation of the study and its implications for them. It will be explained that they may refuse to take part in, or withdraw from

the study without prejudice to their future care and treatment. Written informed consent will be obtained from all patients.

13.4 Health Research Ethics Committee

The protocol, patient information sheet and informed consent form must be approved by the Human Research Ethics Committee in compliance with applicable regulatory requirements. A copy of the written approval/advice must be sent to the Kinghorn Cancer Centre and the Garvan Institute of Medical Research, which should outline the documents approved (protocol, information sheets, informed consent forms and questionnaires) and the version and date of approval. A copy of the signed informed consent should be sent to the Clinical Trials office at the Kinghorn Cancer Centre of St Vincent's Hospital Sydney.

13.5 Confidentiality, Data handling and Record keeping

Each participant will be allocated a study number, which will be used in the database. All information collected at pre-specified time points will be de-identified and stored in the study database. All safety measures have been put in place to ensure that the confidentiality of the participant's information is maintained, including removal of identifying information, the use of unique study numbers and adherence to strict guidelines regarding data transfer, storage and access.

Location of stored data- Electronically stored data will be kept on a dedicated lap-top computer and secured with password login known only to the investigators. A back up of electronic data will be kept on a dedicated external hard drive and stored in a locked cabinet within the principle investigator's department.

Format of stored data- Participant data will be re-identifiable by linking the participants study number assigned at enrolment to their Medical Record Number (MRN). Patient's identity will be held at each site where a patient identification log will be completed.

Arrangements for security of stored data- Security of stored data will be managed by the principle investigator. All electronically stored data will be password protected and portable devices will be locked in secure cabinets within the investigators department. All paper-based documentation will be stored in a locked cabinet in the principle investigators department.

Duration data will be stored- Research data will be retained for five years from the date of publication.

Method of destruction of data- Paper based data will be destroyed by moving to a confidential waste bin. Electronic data will be erased from computer memory.

13.6 Protocol amendments

Changes and amendments to the protocol can only be made by the Study Management Committee. Approval of amendments by the HREC is required prior to their implementation. In some instances, an amendment may require a change to a consent form. The Investigator must receive approval/advice of the revised consent form prior to implementation of the change. The investigator should not implement any changes to, or deviations from, the protocol except where necessary to eliminate immediate hazard(s) to study subject(s).

14. REFERENCES

1. FDA. FDA label on Prometrium 2009 [updated June 2009; cited 2015 28/12/2015]. Available from: http://www.accessdata.fda.gov/drugsatfda_docs/label/2009/019781s013lbl.pdf.
2. Wilkinson PM, et al. Tamoxifen (Nolvadex) therapy-rationale for loading dose followed by maintenance dose for patients with metastatic breast cancer. Cancer Chemother Pharmacol. 1982;10:33-5.
3. Australia C. Breast Cancer Statistics 2015 [cited 2015 30/12/2015]. Available from: http://canceraustralia.gov.au/affected-cancer/cancer-types/breast-cancer/breast-cancer-statistics.
4. Cancer Genome Atlas N. Comprehensive molecular portraits of human breast tumours. Nature. 2012;490(7418):61-70.
5. Bardou VJ*, et al.* Progesterone receptor status significantly improves outcome prediction over estrogen receptor status alone for adjuvant endocrine therapy in two large breast cancer databases. J Clin Oncol. 2003;21(10):1973-9.
6. Purdie CA*, et al.* Progesterone receptor expression is an independent prognostic variable in early breast cancer: a population-based study. Br J Cancer. 2014;110(3):565-72.
7. Rossouw JE*, et al.* Risks and benefits of estrogen plus progestin in healthy postmenopausal women: principal results From the Women's Health Initiative randomized controlled trial. JAMA. 2002;288(3):321-33.
8. de Lignieres B*, et al.* Combined hormone replacement therapy and risk of breast cancer in a French cohort study of 3175 women. Climacteric. 2002;5(4):332-40.
9. Fournier A*, et al.* Breast cancer risk in relation to different types of hormone replacement therapy in the E3N-EPIC cohort. Int J Cancer. 2005;114(3):448-54.
10. Espie M*, et al.* Breast cancer incidence and hormone replacement therapy: results from the MISSION study, prospective phase. Gynecol Endocrinol. 2007;23(7):391-7.
11. Fournier A*, et al.* Unequal risks for breast cancer associated with different hormone replacement therapies: results from the E3N cohort study. Breast Cancer Res Treat. 2008;107(1):103-11.
12. Schneider C*, et al.* Risk of gynecological cancers in users of estradiol/dydrogesterone or other HRT preparations. Climacteric. 2009;12(6):514-24.
13. Birrell SN, et al. Medroxyprogesterone acetate therapy in advanced breast cancer: the predictive value of androgen receptor expression. J Clin Oncol. 1995;13(7):1572-7.
14. Ingle JN*, et al.* Randomized clinical trial of megestrol acetate versus tamoxifen in perimenopausal or castrated women with advanced breast cancer. Am J Clin Oncol. 1982;5(2):155-60.
15. Muss HB*, et al.* Megestrol acetate versus tamoxifen in advanced breast cancer: 5-year analysis--a phase III trial of the Piedmont Oncology Association. J Clin Oncol. 1988;6(7):1098-106.
16. Abrams J*, et al.* Dose-response trial of megestrol acetate in advanced breast cancer: cancer and leukemia group B phase III study 8741. J Clin Oncol. 1999;17(1):64-73.
17. Bines J*, et al.* Activity of megestrol acetate in postmenopausal women with advanced breast cancer after nonsteroidal aromatase inhibitor failure: a phase II trial. Ann Oncol. 2014;25(4):831-6.
18. Germain P*, et al.* Overview of nomenclature of nuclear receptors. Pharmacol Rev. 2006;58(4):685-704.
19. Mohammed H, et al. Progesterone receptor modulates ERalpha action in breast cancer. Nature. 2015;523(7560):313-7.
20. Dowsett M*, et al.* Prognostic value of Ki67 expression after short-term presurgical endocrine therapy for primary breast cancer. J Natl Cancer Inst. 2007;99(2):167-70.
21. Dowsett M*, et al.* Assessment of Ki67 in breast cancer: recommendations from the International Ki67 in Breast Cancer working group. J Natl Cancer Inst. 2011;103(22):1656-64.
22. Hitchcock CL, Prior JC. Oral micronized progesterone for vasomotor symptoms--a placebo-controlled randomized trial in healthy postmenopausal women. Menopause.

2012;19(8):886-93.
23. Shanghold MM et al. Factors associated with withdrawal bleeding after administration of oral micronized progesterone in women with secondary amenorrhea. Fertil Steril. 1991;56(6):1040-7.
24. Byrne MJ*, et al.* Medroxyprogesterone acetate addition or substitution for tamoxifen in advanced tamoxifen-resistant breast cancer: a phase III randomized trial. Australian-New Zealand Breast Cancer Trials Group. J Clin Oncol. 1997;15(9):3141-8.
25. Gill PG*, et al.* Randomized comparison of the effects of tamoxifen, megestrol acetate, or tamoxifen plus megestrol acetate on treatment response and survival in patients with metastatic breast cancer. Ann Oncol. 1993;4(9):741-4.

WinPRO Statistical analysis plan

14.6.2024 Alan Coates

The WinPRO study is a multi-centre three-arm randomized “window of opportunity” trial comparing the short term pre-operative effect of three endocrine therapy options.

Arm 1 letrozole

Arm 2 letrozole plus prometrium

Arm 3 tamoxifen plus prometrium

Primary endpoint

Change in Ki67 percent staining from baseline (Pre) to two weeks (Post), measured as the percent proportional reduction 100*(Pre-Post)/Pre.

The main comparison is between Arm1 and Arm2: this will be tested at the 0.05 level.

The distribution of the proportional reduction by treatment will be tested for deviation from normality. If there is substantial non-normality a non-parametric Wilcoxon test will be used, otherwise a two-sample t-test.

The change in Ki67 will be displayed using Spider plots showing the Pre and Post values for each patient, and as a Waterfall plot of the proportional reduction for each patient in each treatment group

Secondary Endpoints

The other two comparisons, Am1 v Arm 3 and Arm2 v Arm3, will be reported as exploratory using the same graphical presentation but without formal statistical testing.

Complete cell cycle arrest (CCCA) will be defined as a Post Ki67 <= 2.7% following Giltnane JM 2017 (1) and Hurvitz SA 2023 (2). This will be tabulated by treatment and (for the comparison of Arm1 v Arm2) tested by Chi-squared and Fisher’s exact tests

Changes in expression of estrogen, progesterone and androgen receptors will be assessed using H-scores for each receptor before and after treatment. Again the main comparison will be between Arm 1 and Arm 2, with other comparisons being exploratory.

Statistical Software

All analyses will be performed using SAS 9.4 (SAS Institute Inc, Cary, NC, USA).

References

1. Giltnane JM, Hutchinson KE, Stricker TP, Formisano L, Young CD, et al. 2017. Genomic profiling of ER(+) breast cancers after short-term estrogen suppression reveals alterations associated with endocrine resistance. *Sci Transl Med* 9
2. Hurvitz SA, Bardia A, Quiroga V, Park YH, Blancas I, et al. 2023. Neoadjuvant palbociclib plus either giredestrant or anastrozole in oestrogen receptor-positive, HER2-negative, early breast cancer (coopERA Breast Cancer): an open-label, randomised, controlled, phase 2 study. *Lancet Oncol* 24:1029-41
